# Swine influenza viruses in Northern Vietnam in 2013–2014

**DOI:** 10.1038/s41426-018-0109-y

**Published:** 2018-07-02

**Authors:** Eugénie Baudon, Daniel K. W. Chu, Dao Duy Tung, Pham Thi Nga, Hoang Vu Mai Phuong, Nguyen Le Khanh Hang, Le Thi Thanh, Nguyen Thanh Thuy, Nguyen Cong Khanh, Lê Quynh Mai, Nguyen Viet Khong, Benjamin J. Cowling, Marisa Peyre, Malik Peiris

**Affiliations:** 1 WHO Collaborating Centre for Infectious Disease Epidemiology and Control, School of Public Health, The University of Hong Kong-Hong Kong Special Administrative Region, Hong Kong, China; 2 Animal and Integrated Risk Management Research Unit (AGIRs), French Agricultural Research Center for International Development (CIRAD), Montpellier, France; 3 National Institute of Veterinary Research, Hanoi, Vietnam; 4 National Institute of Hygiene and Epidemiology, Hanoi, Vietnam

## Abstract

Swine are an important intermediate host for emergence of pandemic influenza. Vietnam is the largest swine producer in South East Asia. Systematic virological and serological surveillance of swine influenza viruses was carried out in Northern Vietnam from May 2013 to June 2014 with monthly sampling of pigs in local and large collective slaughterhouses and in a live pig market. Influenza A seroprevalence in the local slaughterhouses and in the large collective slaughterhouse was 48.7% and 29.1%, respectively. Seventy-seven influenza A viruses were isolated, all from the large collective slaughterhouse. Genetic analysis revealed six virus genotypes including H1N1 2009 pandemic (H1N1pdm09) viruses, H1N2 with H1 of human origin, H3N2 and H1N1pdm09 reassortants, and triple-reassortant H3N2 viruses. Phylogenetic analysis of swine and human H1N1pdm09 viruses showed evidence of repeated spill-over from humans to swine rather than the establishment of H1N1pdm09 as long-term distinct lineage in swine. Surveillance at the large collective slaughterhouse proved to be the most efficient, cost-effective, and sustainable method of surveillance for swine influenza viruses in Vietnam.

## Introduction

The 2009 H1N1 influenza A pandemic virus (H1N1pdm09) emerged in Mexico as a reassortant virus originating in swine^1,2^. This re-enforced the concept that swine are a “mixing vessel” for avian and human viruses and an intermediate host for pandemic emergence and highlighted the importance of influenza surveillance in swine^3,4^. As the H1N1pdm09 spread in humans worldwide, the virus repeatedly transmitted back to swine leading to several reassortants with local swine viruses^5,6^. Reverse zoonosis from humans to swine appears to take place more readily than zoonotic transmission from swine and thus swine could act as a reservoir of some human viruses or virus genes (as reassortants) long after their disappearance in the human population^7–9^. Zoonotic transmission of a swine H3N2 virus containing the matrix gene from H1N1pdm09, designated as H3N2 variant (H3N2v), has been detected in humans in the USA in 2011, associated with exposure to pigs at agricultural fairs^10^.

South East Asia and China are hotspots for the emergence of infectious diseases due to demographic, social, agricultural, and environmental factors^11^. The Eurasian avian swine and North American Triple Reassortant (TRIG) virus lineages have been introduced to this region through importation of live pigs, leading to generations of a diversity of swine influenza viruses (SIV) through reassortment^12,13^. Avian influenza viruses have also transmitted to swine, probably due to farming practices in which poultry and pigs are raised together in low biosecurity settings, but these avian viruses have not got established in the swine population^12^. H9N2, H5N1, and H7N9 are frequently detected influenza subtypes in poultry, often raised in close proximity to swine, raising concerns of generating novel reassortants with endemic swine viruses because of the potential role of swine as a “mixing vessel” for the avian and human influenza virus gene pools^14,15^. Vietnam has a large swine population and avian influenza viruses such as the highly pathogenic avian influenza (HPAI) H5N1 are endemic in ducks and poultry. Pigs and poultry are often raised in familial multi-species farms with low biosecurity^15–20^ posing a risk for influenza reassortments in pigs. Therefore, influenza surveillance in swine is a priority in this region. While human and avian (HPAI) influenza surveillance is conducted in Vietnam^21,22^, swine influenza (SI) surveillance has had less attention, especially because SIV does not cause significant morbidity and mortality in swine and is thus perceived to be of low economic impact in animal husbandry. Disease surveillance systems are often costly and there is a need to develop cost-effective and sustainable surveillance especially in resource scarce countries^23,24^.

The focus of the present study was the development of sustainable surveillance strategies for SI in North Vietnam. Surveillance protocols were designed and tested in the field for 14 months in 2013–2014, and their cost-effectiveness was evaluated. The main objectives of the surveillance were to characterize the SIV circulating in Northern Vietnam and to describe SI epidemiological features such as seroprevalence of influenza A and different strains in the farming sectors, dynamic of infection including age of infection and potential seasonality.

## Results

### Sample description

Virological and serological sampling was performed from May 2013 to June 2014. The study sites were a collective slaughterhouse in Hanoi (nasal swabs = 2051; sera = 964 collected), three local slaughterhouses (nasal swabs = 681; sera = 677 collected) and a weaner market (nasal swabs = 703; sera = 609 collected) in Hung Yen province. The ages of the pigs sampled were 4 to 6 months old in slaughterhouses (no individual data was recorded) and 1.5 to 3 months old (mean = 2.3 months) at the weaner market. In the collective slaughterhouse, based on official records, slaughtered pigs were sourced from 18 provinces, with pigs originating from 5 to 10 provinces on a single field visit (mean = 9 provinces). Overall, 88% of the pigs originated from the Red River Delta (RRD) (40% from Hanoi, 18% from Ha Nam), 8% from Northern provinces and 4% from Central provinces. Based on trader questionnaires, it was possible to trace the province of origin for each pig for 58% of the sampled pigs (Supplementary Figure S1). The large company sector provided 42% of the sampled pigs, 20% arose from the familial husbandry sector and 38% had no data recorded. From the previous network analysis, it is likely that the familial farms were predominantly large farms^25^.

In local slaughterhouses, 93% of the pigs sampled came from Hung Yen province, mainly the Van Giang district (Supplementary Figure S1). The pigs originated mainly from the familial sector (88%) including 53% from large farms and 35% from small farms, and rarely from large companies (3%; with 9% of missing data). Pigs in the weaner market originated mainly from Bac Ninh in the RRD (67%) and the remainder from other nearby provinces (Supplementary Figure S1). Almost all these pigs originated from small familial farms (97%) while 3% originated from large familial farms.

### Overall results of virus isolation

All nasal swab samples from the weaner market and the local slaughterhouses were negative for SIV while 77 SIV were isolated at the collective slaughterhouse (3.8% virus isolation rate) (Supplementary Table S2). Although higher isolation rates were observed in August 2013, February, March and May 2014, (Supplementary Table S3), no clear seasonality could be defined. Virus isolation positive pigs originated from large companies (21 pigs) and from familial farms (13 pigs); source data were missing for remaining isolates. All three virus subtypes (H1N1, H1N2, H3N2) were detected in pigs from familial and company farms. The isolation rate in pigs originating from companies (2.4%) did not significantly differ from those from familial farms (3.2%) (*χ*^2^ = 0.42, p = 0.52). Pigs with positive virus isolates originated from Hanoi (17% of the positive isolates), Ha Nam (12%), and from three other provinces in the RRD (8%), with missing data for remaining isolates.

**Fig. 1:**
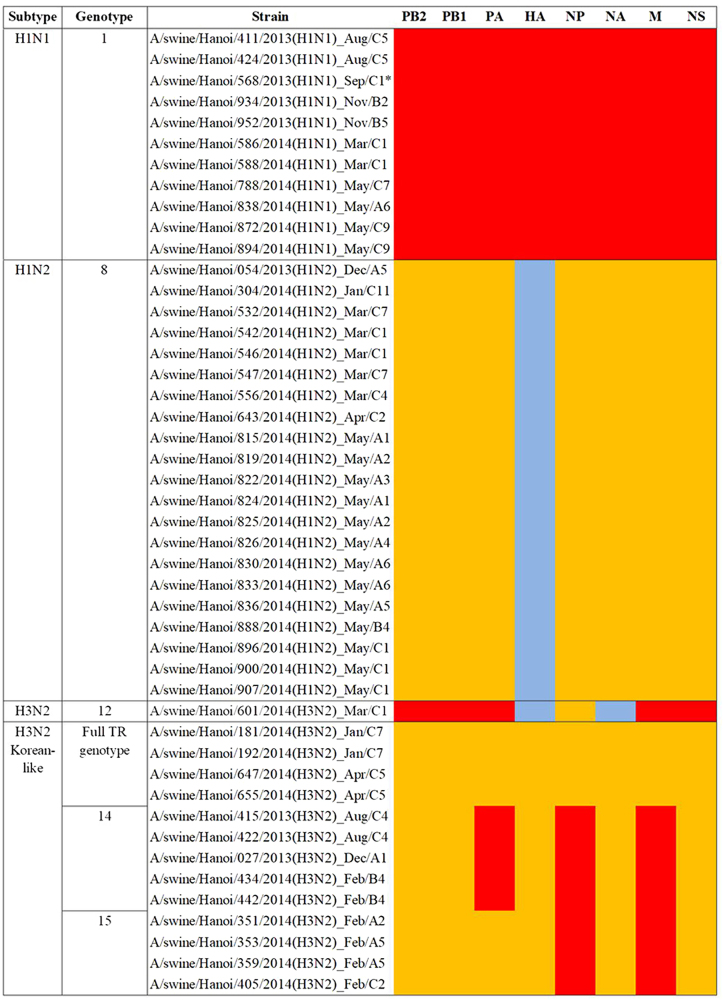
Genotypes of the viruses isolated in Hanoi in 2013–2014. Genotype numbers were defined by Takemae et al.^28^ Lineages: red: H1N1 Pandemic 2009; yellow: Triple-reassortant; blue: human-like

Four viral lineages were identified based on HA and NA sequencing. These were (a) H1N1 of pandemic 2009 origin (H1N1pdm09) (*n* = 16), (b) H1N2 viruses (*n* = 35) with H1 related to seasonal human and SIV from Southern Vietnam (Binh Duong, ‘BD’)^26^ and N2 of triple-reassortant (TR) origin not related to Vietnamese SIV, (c) H3N2 viruses (*n* = 25) with HA and NA related to Korean TR viruses, and (d) H3N2 virus (*n* = 1) with HA and NA derived from human seasonal influenza viruses related to SIV from Binh Duong in Southern Vietnam (Supplementary Table S2). Viruses from each of these groups representing different regions of the phylogenetic tree were selected for full genome sequencing, and six genotypes were identified (Fig. 1).

**Fig. 2:**
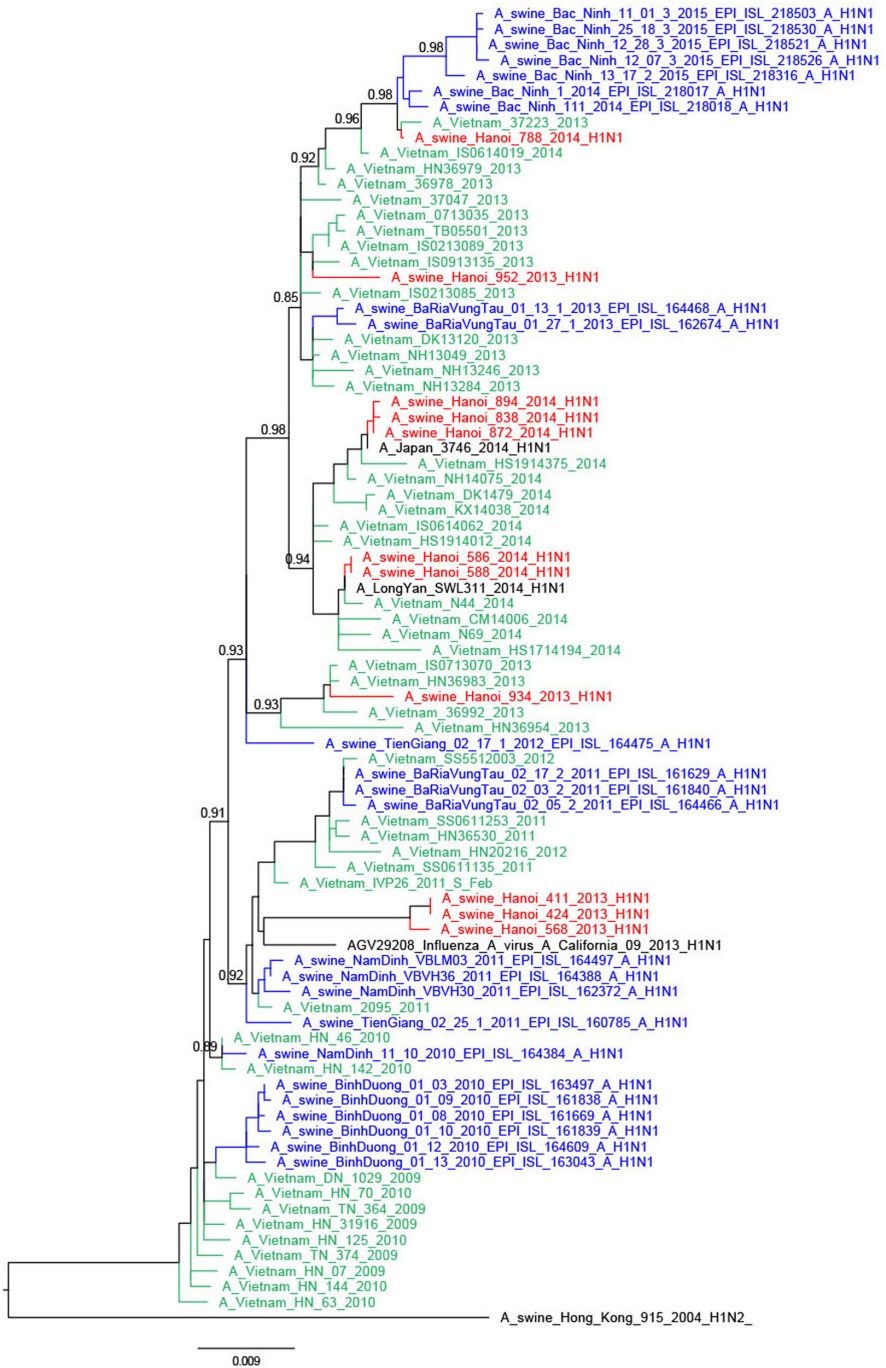
Phylogenetic tree for the full-length hemagglutinin of H1N1pdm09 viruses isolated in a slaughterhouse in Vietnam in 2013–2014. The trees were constructed with PhyML. Branch support aLRT statistics were shown at major nodes with values larger or equal to 0.8. GenBank accession numbers of retrieved sequences are indicated. Red: sequences from our study; blue: sequences from Takemae et al.^28^; green: viruses from Vietnam from other studies including H1N1pdm09 from NIHE; black: other sequences from GenBank

**Fig. 3:**
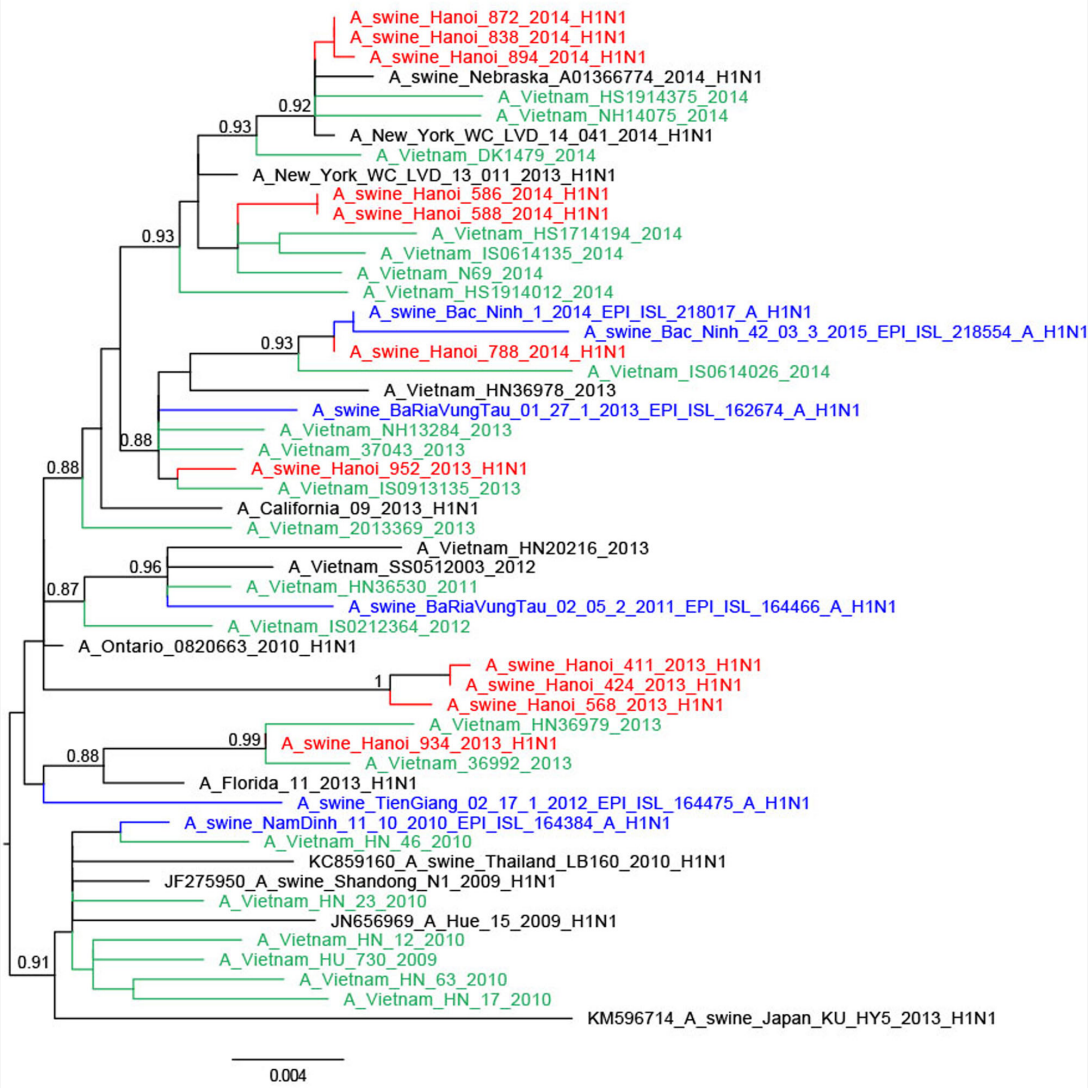
Phylogenetic tree for the full-length neuraminidase of H1N1pdm09 viruses isolated in a slaughterhouse in Vietnam in 2013–2014. The trees were constructed with PhyML. Branch support aLRT statistics were shown at major nodes with values larger or equal to 0.8. GenBank accession numbers of retrieved sequences are indicated. Red: sequences from our study; blue: sequences from Takemae et al.^28^; green: viruses from Vietnam from other studies including H1N1pdm09 from NIHE; black: other sequences from GenBank

**Fig. 4:**
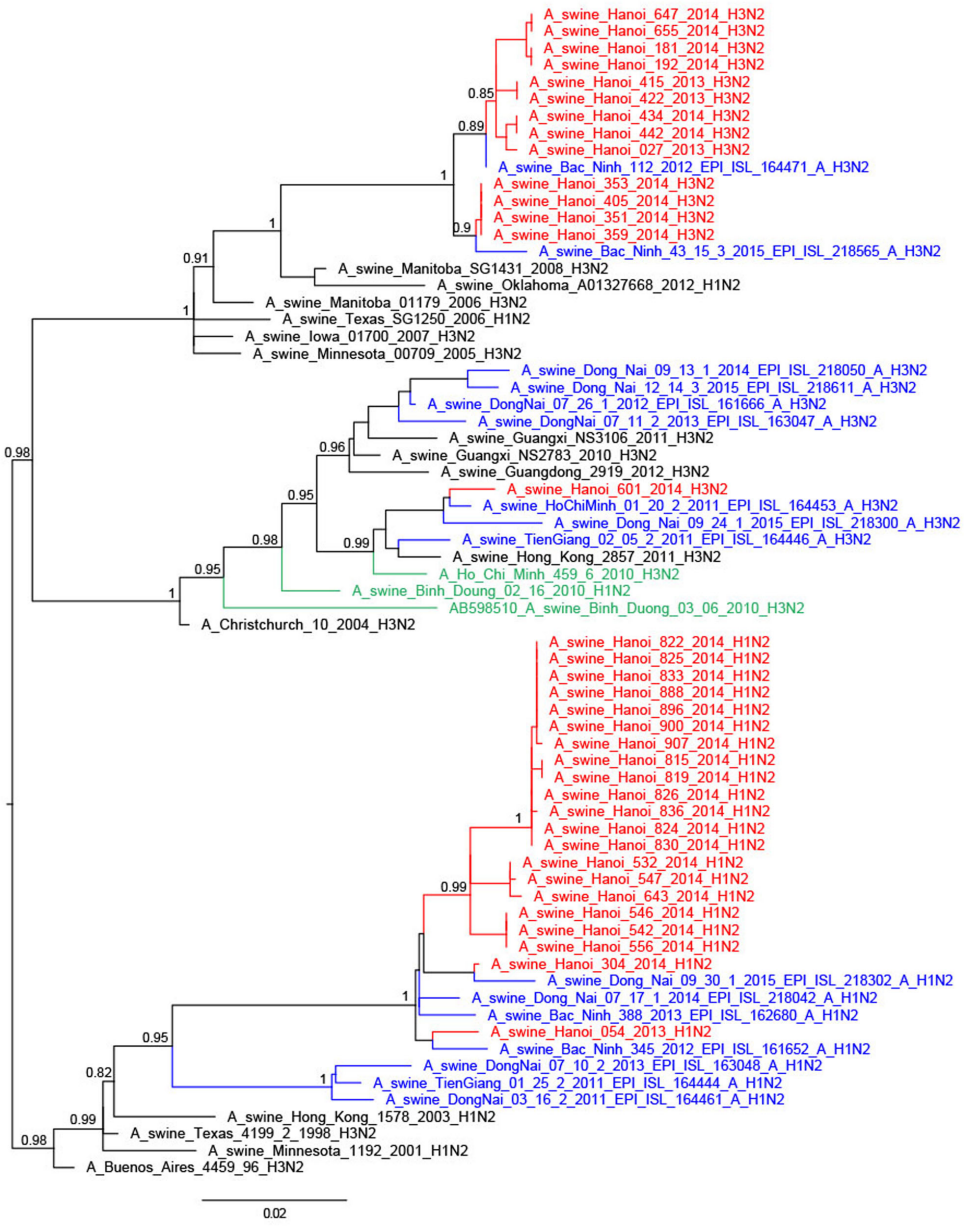
Phylogenetic tree for the full-length neuraminidase of H1N2 and H3N2 viruses isolated in a slaughterhouse in Vietnam in 2013–2014. The trees were constructed with PhyML. Branch support aLRT statistics were shown at major nodes with values larger or equal to 0.8. GenBank accession numbers of retrieved sequences are indicated. Red: sequences from our study; blue: sequences from Takemae et al.^28^; green: viruses from Vietnam from other studies including H1N1pdm09 from NIHE; black: other sequences from GenBank

Eleven of the 16 H1N1pdm09 viruses had full genomes sequenced and all eight gene segments of these were of H1N1pdm09 derivation, including the three A/swine/Hanoi/411/2013-like viruses^27^ (Figs. 2 and 3; Supplementary Figures S4–6 Trees for PB1, PA, and NS genes are available from authors). Twenty-one of the 35 H1N2 viruses had full viral genomes sequenced and all were closely related to other contemporary SIV isolated in Bac Ninh (BN) province in the RRD^28^ and had nearest human H1 ancestral viruses from around 2006 (e.g., A/Hanoi/Q591/2006; H1N1) (Supplementary Figure S7). The NA gene and the 6 internal gene segments were all of TR derivation (Fig. 4; Supplementary Figures S4–6). These H1N2 SIV had clearly established a persistent long-term stable lineage in swine.

**Fig. 5:**
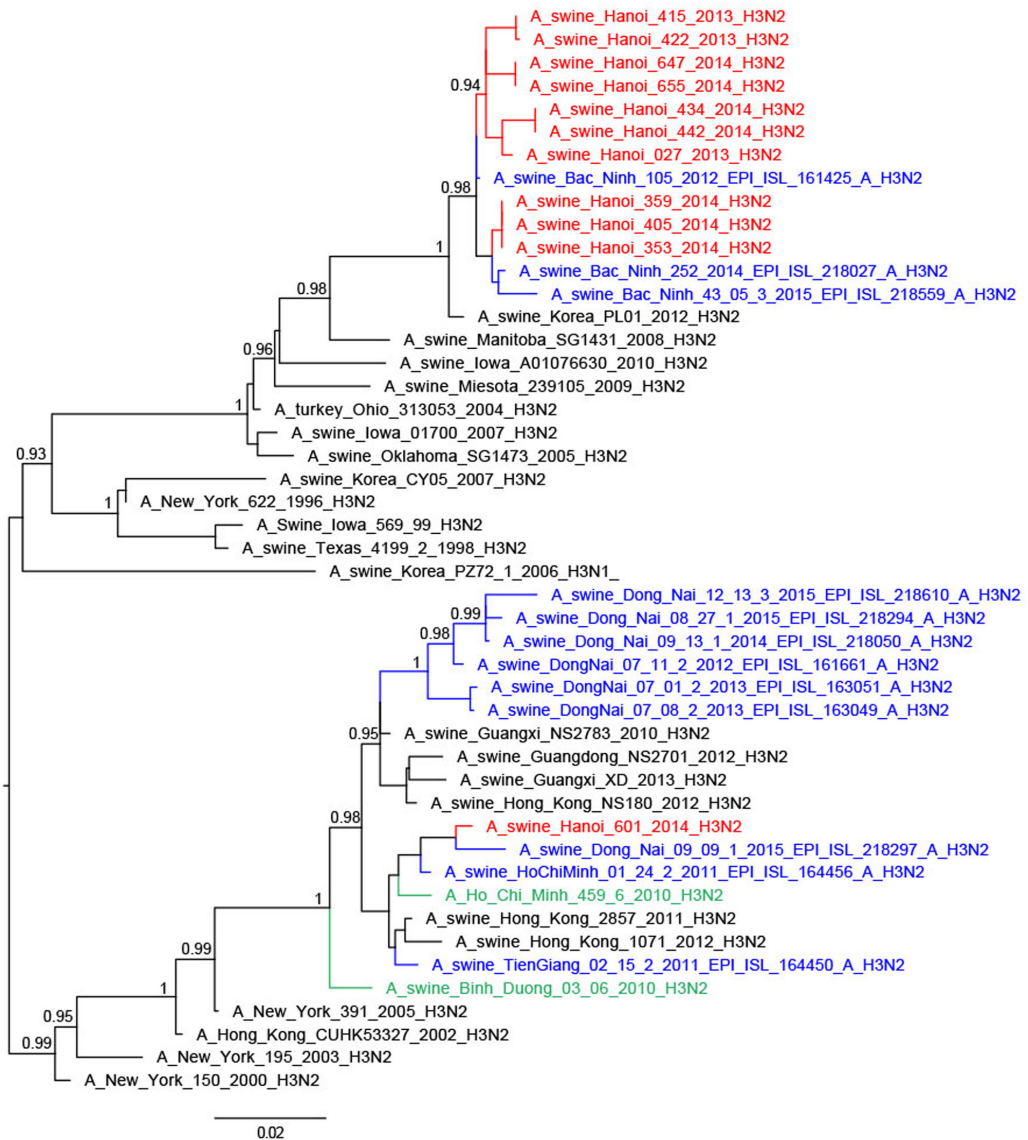
Phylogenetic tree for the full-length hemagglutinin of H3N2 viruses isolated in a slaughterhouse in Vietnam in 2013–2014. The trees were constructed with PhyML. Branch support aLRT statistics were shown at major nodes with values larger or equal to 0.8. GenBank accession numbers of retrieved sequences are indicated. Red: sequences from our study; blue: sequences from Takemae et al.^28^; green: viruses from Vietnam from other studies including H1N1pdm09 from NIHE; black: other sequences from GenBank

Thirteen of the 25 H3N2 TR viruses had full genomes sequenced; they were related to H3N2 TR viruses from South Vietnam, Korea and USA^29^, the nearest ancestral human viruses being H3N2 viruses from around 1996 (A/New York/622/1996)^28,30^ (Fig. 5). Four of these viruses had the NA and internal genes similar to other TR viruses (full TR genome) (Fig. 4; Supplementary Figures S4–6). Four others had M and NP gene segments derived from H1N1pdm09, while another five had PA, M, and NP gene segments of H1N1pdm09 derivation. The A/swine/Hanoi/601/2014 (H3N2) virus with the nearest ancestral HA and NA being H3N2 human viruses from 2004/5 (e.g., A/New York/391/2005)^31^ had NP gene of TR derivation while the other five internal gene segments were of H1N1pdm09 derivation. This virus was similar to viruses previously reported from Vietnam and Southern China^28,31^. No viruses of Eurasian Avian (EA) lineage, an SIV lineage predominant in China, were isolated in this study. The study by Takemae et al.^28^ in Vietnam over the period 2010–2015 also did not find any EA lineage viruses in Vietnam.

Thus, the 46 SIV with full genome sequences could be classified into six genotypes based on the derivation of all eight gene segments (Fig. 1). Five of these genotypes have been previously described in Vietnam in previous publications^27,28^ and the genotype designations from the study by Takemae et al. are denoted in Fig. 1 for ease of comparison. The H1N1 viruses with all the eight segments of pdm09 lineage (genotype 1) have been isolated in Northern and Southern Vietnam. The 21 H1N2 with full genome sequenced (genotype 8) have also been isolated in Northern and Southern Vietnam in 2012–2015. The one BD-like H3N2 isolate A/swine/Hanoi/601/2014 (genotype 12) was previously only reported in Southern Vietnam in 2011–2015^28^. The Korean-like H3N2 isolates from August 2013, December 2013, and February 2014 (genotype 14) were similar to viruses isolated in 2012 in Northern Vietnam while the four ‘genotype 15’ H3N2 isolates from February 2014 were similar to those isolated in 2013-2015 in Northern Vietnam. The four A/swine/Hanoi/181/2014 (H3N2)-like viruses with all eight gene segments of TR derivation have not been previously documented in Vietnam. However, their HA and NA genes cluster very closely with the TR A/Hanoi/415/2013(H3N2)-like viruses (Figs. 4 and 5) with PA, M, and NP of H1N1pdm09 derivation (genotype 14). Thus, it is likely that A/swine/Hanoi/181/2014 (H3N2)-like viruses were the precursors of genotype 14 and 15 viruses, which acquired H1N1pdm09 gene segments via reassortment; the internal genes of H1N1pdm09 lineage for these genotypes were closely related to genes from H1N1pdm09 viruses circulating in humans in Vietnam in 2009 (Supplementary Figures S4–6). These results suggest multiple reassortments between endemic H1N2 and H3N2 swine viruses and between endemic H3N2 swine viruses and H1N1pdm09 human viruses. Among the three reassortants with H1N1pdm09 virus genes, the H1N1pdm09 M gene was the gene present in all three. On the contrary, H1N1pdm09 HA and NA genes were never found in reassortant viruses and were only found when the full H1N1pdm09 gene constellation remained preserved.

### H1N1pdm09 isolates

In order to investigate whether the swine H1N1pdm09 isolates had established a distinct long-term lineage in swine or whether they represented repeated spill-over events from viruses circulating in humans, we obtained HA and NA virus sequence data from the National Institute of Hygiene and Epidemiology (NIHE), Hanoi. They had reported that there were three main waves of H1N1pdm09 infection in humans in Vietnam up to the end of 2013^32^. The first wave started with the first introduction of the pandemic to Vietnam at the end of May 2009 and lasted until early 2010; the second wave occurred from November 2010 to April 2011 with a secondary peak from June to November 2011, and the third wave started early January 2013 and lasted until around September 2013. In 2012, isolation of human H1N1pdm09 viruses was only occasional. Fewer cases were then detected after 2013, a fourth and fifth small waves occurred from February to April 2014 and from March to June 2015, respectively^33^.

Within the phylogenetic trees, the swine H1N1pdm09-like viruses of 2013 and 2014 were not monophyletic but interspersed with the chronologically related human H1N1pdm09 viruses of the same or adjacent year suggesting separate spill-over events of H1N1 viruses from humans to swine, each spill-over not establishing stable long-term virus lineages in swine (Figs. 2 and 3). The HA and NA of 934/2013 clustered with Vietnamese human viruses from April 2013 while the HA and NA of 952/2013 were most related to Vietnamese human viruses from March 2013, both related to the third wave of H1N1pdm09 circulation in humans in Vietnam. The swine isolates from March 2014 (586/2014, 588/2014) were closely related to human viruses such as A/Vietnam/N44/2014-N/Feb from February 2014 (fourth wave). Three isolates from May 2014 (838/2014, 872/2014, and 894/2014) were related to A/Vietnam/37271/2014-N/Feb, a human H1N1pdm virus from Vietnam from February 2014 (fourth wave) and other viruses from 2014 from around the World. The fourth isolate from May 2014 (788/2014) clustered with contemporary Vietnamese human viruses from November 2013 for HA and January 2014 viruses (fourth wave) for NA. Comparison of the dates of isolation of the most closely related human virus and the swine isolate varied from 1 to 7 months.

An exception was three H1N1pdm09 SIV from 2013. A/swine/Hanoi/411/2013 and A/swine/424/2013 viruses from August 2013 and A/swine/Hanoi/568/2013 virus from September had a long branch distinguishing them from the human H1N1pdm09 backbone and had a sister clade relationship with a human H1N1pdm virus isolated in California in 2013 (A/California/09/2013), the closest viruses from Vietnam being those from 2011–12, probably corresponding to viruses from the second wave of H1N1pdm09 circulation in humans in Vietnam^32^. It is possible that unsampled genetic diversity of human H1N1pdm viruses in Vietnam contributed to the lack of closer human viruses from Vietnam^27^.

As with the phylogenetic trees for the HA and NA, the internal gene segments of the H1N1 viruses were also not monophyletic and were interspersed between human H1N1 virus gene sequences (Supplementary Figures S4–6). Taken together, these results suggest that the swine H1N1pdm viruses originated from separate spillovers events from human H1N1pdm viruses rather than as a result of H1N1pdm establishing a continuous sub-lineage within swine.

### Potential transmission at the slaughterhouse

Isolates from a same subtype, isolated in the same month and from the same pen were nearly identical in the phylogenetic analysis for the eight gene segments (e.g., H1N1 411/2013_Aug/C5 and 424/2013_Aug/C5; H1N2 542/2014_Mar/C1 and 546/2014_Mar/C1; H3N2 434/2014_Feb/B4 and 442/2014_Feb/B4) (Supplementary Figures S4–6). On the contrary, some isolates which only differed by the pen of sampling were genetically distant enough to suggest they originated from different farms (e.g., H1N1 934/2013_Nov/B2 and 952/2013_Nov/B5; H1N2 907/2014_May/C12 and other May isolates; H3N2 Feb/B4 isolates and other February isolates). Finally, sometimes isolates from different pens were nearly identical (e.g., H1N1 May/C9 and May/A6 isolates; H1N2 Mar/C1 and Mar/C4 isolates; H3N2 Feb/A2, Feb/A5, and Feb/C2 isolates). In this instance, there were two hypotheses: pigs from the same farm were dispatched into different pens at the slaughterhouse or SIV transmission occurred at the slaughterhouse infecting pigs from different farms. Either or both scenarios may well have occurred. Interestingly, however, in May, nearly identical H1N2 viruses were isolated from eight different pens (A1, A2, A3, A4, A5, A6, B4, C11, C12), suggesting that SIV transmission may have occurred at the slaughterhouse. However, transmission at the slaughterhouse seemed unlikely to occur as a large number of pigs arrive by trucks a few hours before slaughtering, and on each sampling day, the number of incoming pigs recorded in official records was close to the total capacity of the slaughterhouse. Therefore, most pigs were likely slaughtered the same day and cross-infection between pigs would be unlikely as viral shedding has been shown to start from one to 3 days post infection^34^. Also transmission during transportation was probably limited as the provinces of origin were within a driving time of 12 h; it was unknown whether pigs from several farms were transported together. The data on pig origins were not sufficiently detailed to be used in this analysis. Our results demonstrate the value and feasibility of molecular epidemiology to provide insights into the transmission pathways of SIV. More detailed data on pig origin and time spent by pigs in transportation and at the slaughterhouse together with environmental sampling in slaughterhouses would be very useful to understand the roles of contaminated surfaces, and potentially aerosols, in the transmission of SIV during transportation and/or in slaughterhouses as surfaces and aerosols have been shown to contain significant levels of SIV in barns with SIV outbreaks^35^.

### Serological evidence of virus infection by different strains

The sera were screened with an influenza A reactive ELISA detecting antibodies against the nucleoprotein (NP) of influenza A viruses and positive sera were tested with a panel of SIV antigens based on viruses isolated during this study and also a European Avian lineage influenza virus isolated in China. Only sera that were positive in HI tests were regarded as true positives. A total of 68% (628/927) of the ELISA positive sera were positive by HI test. The overall HI seroprevalence was 29.1% (min = 12.9%, max = 47.1%) at the collective slaughterhouse, 48.7% (min = 14.3%, max = 80.6%) in local slaughterhouses and 2.8% (min = 0%, max = 15.2%) at the weaner market. At the weaner market, sero-positive pigs were 1.5–3 months old. Maternal antibodies often disappear around 2 months of age^36^, thus it was not possible to assess with certainty if the sero-positivity detected at the market was due to infection or maternal passive immunity. However, the overall low prevalence level observed suggested that in most cases maternal antibodies had already waned or were not present, and natural infection had not yet occurred. In Vietnam, influenza vaccines are not used in swine. Pigs at the market originated exclusively from familial farms, mainly small farms.

**Fig. 6:**
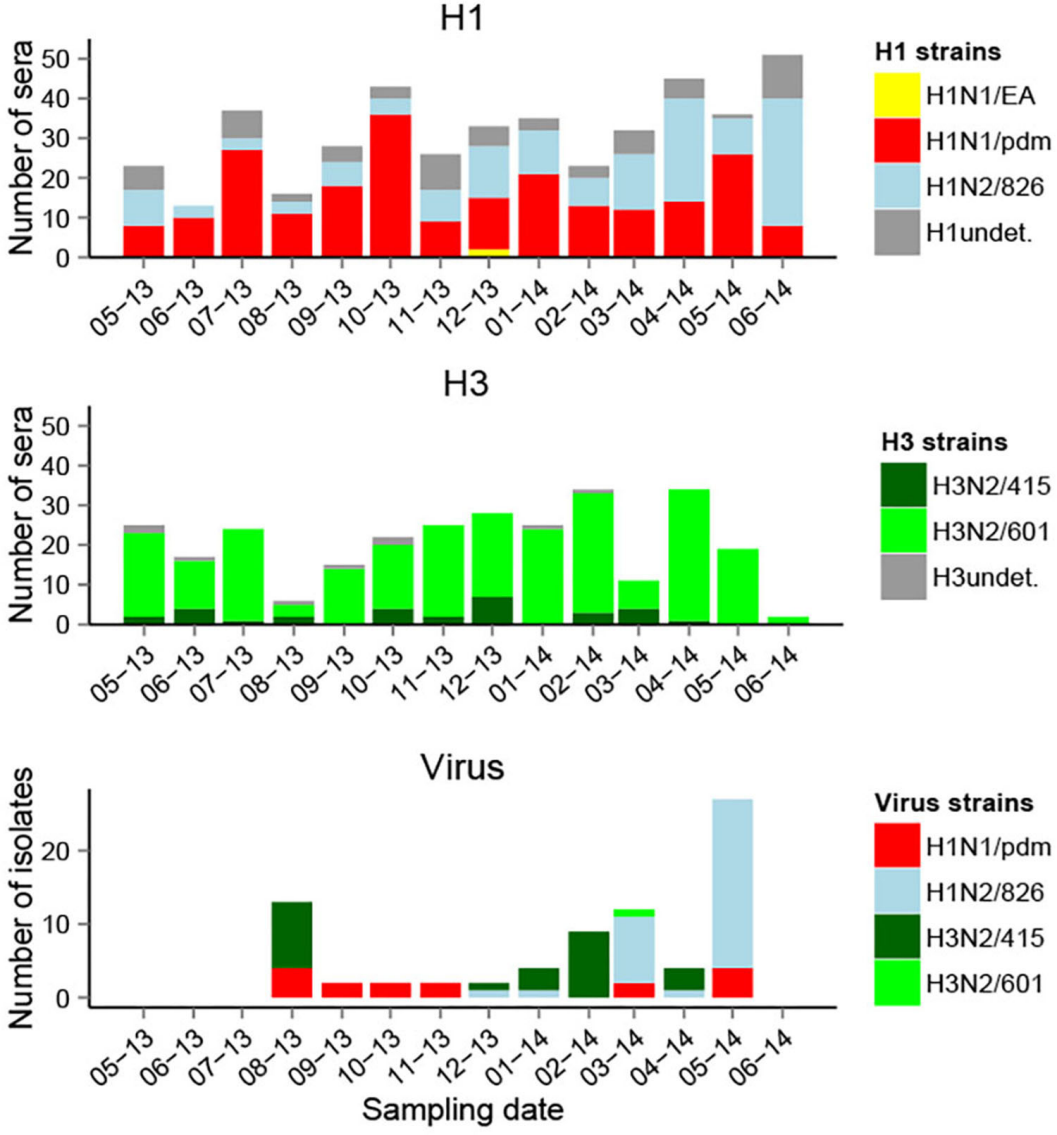
Serological and virological distributions of different influenza A strains across time in slaughterhouses. Undet. undetermined strain, i.e., HI titer positive to several H1 or H3 strains with a titer difference < 4-fold. Viruses were isolated at the collective slaughterhouse

Taking into account all HI test results, H1N1/pdm (13.5% seroprevalence, geometric mean HI titer g = 87) and Binh Duong-like H3N2/601 (11.7% seroprevalence, g titer = 161) showed the highest overall HI seroprevalence across locations (Table 1). Only these two strains were detected by HI test at the weaner market. The overall seroprevalence for H1N2/826 was 8.6% (g titer = 107), and seroprevalence for Korean-like H3N2/415 (1.9% seroprevalence, g titer = 69) and H1N1/EA (1.7% seroprevalence, g titer = 42) were much lower overall. Most H1N1/EA positive sera had fourfold higher antibody titers for H1N1/pdm (Fig. 6, Supplementary Figure S8) and it is likely that these H1N1/EA reactive antibodies reflect cross-reaction. This is in agreement with the absence of EA virus isolates. Sero-positive sera were present in every sampling month against all other strains isolated at the collective slaughterhouse, except for the Korean-like H3N2/415. The high seroprevalence level of H1N1/pdm across sampling time and locations was consistent with frequent isolation of H1N1pdm09 viruses. On the contrary, only one Binh Duong-like H3N2/601 virus was isolated in the slaughterhouse in March 2014, despite high seroprevalence to this strain. Korean-like H3N2/415 and H1N2/826 viruses were frequently isolated from August 2013 and from December 2013, respectively, in the collective slaughterhouse, but H3N2/415 showed low seroprevalence levels, and H1N2/826 had variable seroprevalence levels across time. By sampling design, only half of the pigs tested by virus isolation were also tested serologically, and therefore only 39 of the 77 virus positive pigs had also been tested by serological tests. Eight pigs positive by virus isolation were also positive by HI test. Only one was positive by both analyses for the same strain (H1N2/826, HI titer = 80), two pigs were positive by both analyses for a same HA subtype but a different strain (HI titers = 160-320), and five were only positive for a different HA subtype (HI titers = 20–160).

**Table 1:** Hemagglutination inhibition seroprevalence for the different strains of influenza A across locations

HI strains	HI seroprevalence % (number of sera)			
	Collective slaughterhouse (*N* = 964)	Local slaughterhouses (*N* = 677)	Market (*N* = 609)	Overall (*N* = 2250)
H3N2/415	3.4 (33)	1.5 (10)	0 (0)	1.9 (43)
H3N2/601	10.1 (97)	23.6 (160)	1.1 (7)	11.7 (264)
H1N1/pdm	14.2 (137)	23.2 (157)	1.6 (10)	13.5 (304)
H1N2/826	10.9 (105)	13.1 (89)	0 (0)	8.6 (194)
H1N1/EA	2.8 (27)	1.6 (11)	0 (0)	1.7 (38)
Total HI positive	29.1 (281)	48.7 (330)	2.8 (17)	27.9 (628)

All HI test results were included regardless of titer differences between strains of same subtype

In univariate analyses using all HI test results from slaughterhouses, all strains showed significant differences according to the province of origin and according to the farm category (H1N1/EA being the exception, but probably reflecting cross-reactive antibody). In final models (Table 2), the circulation of strains was significantly different across the farm categories for H3N2/415 (higher circulation in companies, OR = 3.6) and H1N1/pdm (higher circulation in familial farms, OR = 0.55), and across provinces for H3N2/601 (lower circulation in Hanoi compared to Hung Yen, OR = 0.15) and H1N1/EA (higher in Hanoi, OR = 3.4). Sero-positivity to H1N1/EA (OR = 25.6 for factor “positive to another H1 strain”) was compatible with cross-reaction. Strain circulation was also positively associated to virus isolation in the same month for H3N2/415 (OR = 2.0), and H1N2/826 (OR = 2.5), indicating a new introduction or an increase in spread, and negatively associated for H3N2/601, indicating this strain was probably not newly circulating. Differences in the circulation between small and large familial farms were also investigated at the local slaughterhouses; such information was not available at the collective slaughterhouse. No significant difference was observed in the circulation of the different strains among these farms, with all *p*-values above 0.2 in univariate analysis (data not shown).

**Table 2:** Logistic regression results for the different influenza A strains

Variables	*β*	SE	OR	95% CI		*p*-value	
				Lower	Upper		
H3N2/415 (AIC: 338.07)							
(Intercept)	−4.315	0.387	0.013	0.006	0.027	0.000	***
Familial farm							
Company farm	1.269	0.573	3.558	1.195	11.331	0.027	*
Unknown farm	1.134	0.645	3.107	0.842	10.698	0.079	
Hung Yen							
Hanoi	0.964	0.608	2.621	0.817	8.837	0.113	
Other	0.074	0.599	1.077	0.317	3.394	0.902	
Unknown	−0.378	0.693	0.685	0.178	2.740	0.585	
No concurrent isolation							
Concurrent isolation	0.690	0.325	1.993	1.046	3.773	0.034	*
H3N2/601 (AIC: 1078.8)							
(Intercept)	−0.465	0.110	0.628	0.505	0.779	0.000	***
Hung Yen							
Hanoi	−1.918	0.307	0.147	0.077	0.259	0.000	***
Other	−0.191	0.198	0.826	0.558	1.212	0.334	
Unknown	−0.456	0.197	0.634	0.428	0.928	0.021	*
Before 1st isolation							
After 1st isolation	−0.632	0.170	0.532	0.379	0.738	0.000	***
H1N2/826 (AIC: 946.59)							
(Intercept)	−1.920	0.173	0.147	0.103	0.204	0.000	***
Familial farm							
Company farm	−0.543	0.285	0.581	0.329	1.009	0.057	
Unknown farm	−0.121	0.307	0.886	0.478	1.598	0.694	
Hung Yen							
Hanoi	−0.224	0.353	0.799	0.393	1.578	0.526	
Other	0.332	0.229	1.394	0.886	2.178	0.147	
Unknown	0.572	0.327	1.771	0.937	3.392	0.081	
Before 1st isolation							
After 1st isolation	0.914	0.180	2.493	1.759	3.572	0.000	***
H1N1/pdm (AIC: 1179.9)							
(Intercept)	−0.636	0.112	0.530	0.425	0.658	0.000	***
Familial farm							
Company farm	−0.601	0.168	0.549	0.393	0.759	0.000	***
Unknown farm	−0.846	0.206	0.429	0.284	0.637	0.000	***
No concurrent isolation							
Concurrent isolation	0.278	0.142	1.321	0.999	1.744	0.050	
H1N1/EA (AIC: 276.55)							
(Intercept)	−6.275	0.769	0.002	0.000	0.007	0.000	***
Hung Yen							
Hanoi	1.221	0.474	3.391	1.310	8.593	0.010	**
Other	1.173	0.437	3.232	1.366	7.726	0.007	**
Unknown	0.549	0.525	1.731	0.579	4.712	0.296	
Not positive to other H1							
Positive to other H1	3.245	0.733	25.650	7.713	159.067	0.000	***

Only sera from slaughterhouses were included *p-value ≤0.05; **p-value ≤0.01; ***p-value ≤0.001

## Discussion

A total of 77 SIV of H1N1, H1N2, and H3N2 subtypes were isolated in the collective slaughterhouse (isolation rate = 3.8%), an isolation rate similar to that reported from surveillance in farms in Southern and Northern Vietnam^28^ and other studies across Asia^37^. We did not isolate any SIV in the weaner market or farms. Interestingly, there was no evidence that these weaner markets were serving as amplifiers and disseminators of virus, in contrast to what has been reported for live poultry markets. Our surveillance in the collective slaughterhouse was therefore pursued further and represents the first ongoing long-term longitudinal surveillance program for SIV in slaughterhouses in Vietnam; previous studies being unsuccessful in isolating viruses in slaughterhouses in Vietnam^38–40^.

All the H1N1 viruses with HA and NA of pandemic lineage had all eight segments derived from H1N1pdm09. It was clear that most of these H1N1pdm09 SIV arose from separate spillovers from humans and were related to different waves of H1N1pdm09 circulation in humans in Vietnam. The isolates from August and September 2013 did not cluster clearly with 2012 or 2013 human viruses from Vietnam and seemed more distant from other human virus H1N1pdm sequences available. They may have circulated for a longer period in swine as full pandemic viruses, as shown in the USA for viruses before 2014^41^ or the longer phylogenetic distance of these viruses may reflect unsampled virus diversity of human viruses circulating in 2012 in Vietnam^32^.

H3N2 TR viruses of the Korean TR lineage were isolated in August 2013 and then regularly from December 2013 to April 2014. Some of these had all 8 gene segments of TR origin while others had acquired M, NP and sometimes PA gene segments of H1N1pdm09 derivation. The overall seroprevalence of this lineage was 1.9%, suggesting this virus was not yet widespread. Based on these observations and on the very high identity to the Korean TR strain, a plausible hypothesis is that this virus has been introduced recently into Northern Vietnam by swine importation from South Korea. Indeed a report from 2006 mentioned that Vietnam did import breeding pigs in the past from various countries including Korea, but also USA, Canada, Thailand, and Belgium^42^, but no recent data on live pig importation from South Korea could be found.

Seasonal human H3N2 viruses are often transmitted to pigs^12^ but few variants get established long-term in the pig population. The BD-like H3N2 variants with HA and NA that originated from human seasonal H3N2 viruses from 2004 to 2005 and internal genes of H1N1pdm09 origin were first detected in swine in Southern Vietnam in 2010–2015^28,30^. Similar viruses with related HA and NA were isolated in Southern China in 2010 and have been found repeatedly since then^31^. These viruses from China and Vietnam formed a monophyletic clade with the 2010 viruses from Vietnam in ancestral relationship. During our current surveillance in Hanoi, only one BD-like H3N2 virus was isolated (March 2014). The NA and internal genes of this isolate were all closely related to genes from BD-like H3N2 swine viruses isolated in Vietnam and Southern China in 2011 and 2012. While the BD-like HA and NA are conserved, multiple reassortants with internal genes of H1N1pdm09 lineage were found as shown by our surveillance and by the surveillance in Southern Vietnam^28^ and China^31^. Serological analyses showed these BD-like H3N2 viruses were widespread in Northern Vietnam (overall seroprevalence = 11.7%) and seemed to be the dominant H3N2 lineage, even though less BD-like H3N2 virus isolates were obtained as compared with TR H3N2 viruses. It is possible that these viruses are endemic and infect swine early in life, thus being less likely to be detected around the time of slaughter. This highlights the importance of coordinated serological studies being carried out in parallel with virological surveillance.

Although H1N1pdm09 internal genes were commonly detected in H3N2 viruses, it is notable that the H1N1pdm09 HA and NA were not found in these reassortant viruses, as also reported in other studies in Vietnam and elsewhere^28^. Although regular spillovers of H1N1pdm09 viruses occur from humans to pigs, these results support the contention that H1N1pdm09 HA and NA do not seem well adapted to long-term persistence in swine^28,41,43^. On the contrary, H1N1pdm09 internal genes appear to be well adapted to swine, these gene segments, especially the M gene segment, being often acquired by other swine influenza lineages and appearing to provide an evolutionary advantage in swine^31^.

The H1N2 viruses with HA derived from human seasonal influenza viruses of 2004/5 and internal gene segments and NA of TR derivation are well established in the Red River Delta. They were detected by virus isolation repeatedly from December 2013 to May 2014, forming a monophyletic lineage and were detected by serological testing from the beginning of the surveillance period in May 2013 with a seroprevalence of 10.9% in the collective abattoir and 13% in the local slaughterhouses. They have also been detected in Northern and Southern Vietnam by others^28^. However, such viruses were not found during surveillance in Southern China^31^.

The EA-H1 lineages were frequently isolated in recent years in Southern China^31^ but such viruses were not detected during our year of surveillance and convincing seroprevalence to these viruses was also lacking, many of the EA-H1 reactive sera likely being due to cross-reaction with H1N1pdm09. Thus we conclude that the EA lineage appeared to be completely absent in Vietnam as also supported by data from previous studies in Vietnam by others as well as ourselves. This suggests movement of SIV from China into Vietnam in the last 10 years is not very common. However, BD-like H3N2 viruses may well have moved in the opposite direction.

The study demonstrated a high circulation of SIV in the pig population in the study area with overall HI seroprevalence of 29.1% in the collective slaughterhouse and 48.7% in the local slaughterhouses (only 2.8% with younger pigs in the weaner market). These observations suggest that SI infection occurs commonly after the age of 3 months following maternal antibody decay as it has been previously reported^44^. However, a previous study performed in Southern Vietnam has been successful in isolating SIV from 1 to 2 months old pigs from a company farm^30^. In slaughterhouses, differences in seroprevalence were observed between farm categories and provinces of origin according to different viral strains, showing a potential heterogeneity of circulation of SIV which could be linked to various trade routes^25^ or contact patterns between humans and swine for H1N1pdm09. However, the high number of missing data on pig origin at the collective slaughterhouse limited the statistical analyses.

Previous surveillance studies in slaughterhouses in Vietnam have not been productive^38–40^. However, slaughterhouse surveillance has been demonstrated to be an efficient means of SIV surveillance in Hong Kong and mainland China^31^. While we found that surveillance in smaller local slaughterhouses was not productive, our surveillance in a large collective slaughterhouse in Hanoi during the period 2013–14 led to the isolation of viruses of four HA lineages (pdmH1N1, human seasonal H1, human seasonal H3, TR H3) and six different genotypes in Northern Vietnam. Farm surveillance in Northern Vietnam by others during the same period detected three of these HA lineages and five distinct virus genotypes^28^. Surveillance in individual farms is logistically more complex, more labor, and resource intensive and requires acceptance from the individual swine farmers, many of whom may be reluctant to participate. Our study suggests that surveillance in large collective slaughterhouses is an efficient, cost-effective, and sustainable means of SIV surveillance. The value of such surveillance would be greatly enhanced if collection of information on the origins of pigs sourced in the slaughterhouse is improved. Moreover, further studies including sampling on slaughterhouse workers, who were not wearing any personal protective equipment, would be useful to assess influenza transmission from swine to humans.

## Materials and methods

### Pilot surveillance protocols

The selection criteria for the area of study were a high pig density, diversity of farming systems (companies, large and small familial farms), and dynamic and diverse trading practices. Based on the results of a previous network analysis^25^, different locations were selected for sampling of nasal swabs and blood from swine. Longitudinal monthly surveillance in different premises for serological and virological detection of SIV was performed from May 2013 to June 2014. In Hanoi, the main collective slaughterhouse processing about 1500 pigs per day was included with the sampling of 150 nasal swabs and 70 sera every month. In Hung Yen province, three local slaughterhouses (20–30 pigs slaughtered per day; 20–30 nasal swabs and sera collected per slaughterhouse per month) and a weaner market (100 pigs sold by market day; 60 nasal swabs and 50 sera collected per month) were selected. The collective slaughterhouse was a large building with 26 pens used by independent traders to slaughter pigs purchased from various farms. On the contrary, each local slaughterhouse was a place privately owned by one trader (usually located near his house) who purchased pigs from farms and slaughtered them. Data on the farm of origin such as farm category and location, the age of animals (market), the pen number (collective slaughterhouse), and the trader’s name (market) were recorded. The farm categories were previously defined within the network analysis^25^, briefly small familial farms were farms with a number < 100 fattening pigs per cycle and < 10 sows, and large familial farms with a number ≥ 100 fattening pigs per cycle or ≥ 10 sows. Company farms were large farms, usually larger than familial farms, with a different legal status. During sampling, there was no traceability system previously in place and the information on origin of pigs relied on the traders’ memory. However, in the collective slaughterhouse, general data on the number of pigs per province of origin slaughtered in a day were recorded by the slaughterhouse staff and made available.

### Virus isolation and sequencing

Sterile plain dry swabs (Copan™, Brescia, Italy) were used for pigs over 2.5–3 months old, and flexible nasopharyngeal flocked swabs (Copan FLOQSwabs™) were used for younger pigs. The swabs were inserted deep (about 10 cm) into the nasal cavity of the pig and then placed into a tube filled with 1 mL of Viral transport Medium (VTM) containing antibiotics and bovine serum albumin. The samples were placed in an icebox after collection and then stored in a refrigerator at 4 °C upon arrival at the NIVR laboratory in Hanoi. The VTM from the swabs were inoculated on Madin Darby Canine Kidney (MDCK) cells for virus isolation the same day. The MDCK cells were grown in 24 well plates in minimum essential medium (MEM) with trypsin (2 µg/mL). Each inoculated well was separated by an empty well in order to avoid cross-contamination. The next day, the medium was replaced with fresh culture medium. The cells were observed for cytopathic effect (CPE) on day 3 or 4 post-inoculation (DPI) and culture supernatant from each well was harvested for testing by hemagglutination test (HA). When CPE and/or HA was positive, a second passage was carried out. Suspected positive cultures were tested using a rapid antigen detection test Directigen™ EZ Flu A+B (Becton, Dickinson and Company, New Jersey, USA). Positive virus isolates were stored at −80 °C.

Nucleic acid extraction was performed from MDCK cell culture isolates using QIAamp Viral RNA Mini Kit (Qiagen) according to instructions from the manufacturer. Viral RNA was reverse transcribed, cDNA of the HA and NA was PCR amplified and partial sequences determined using Sanger sequencing. A multiplex RT-PCR for determining the origin of virus internal genes^45^ was carried out. These data were used to select isolates for full-length sequencing of all eight gene segments using universal primers of influenza A viruses^46^ and next-generation sequencing (NGS). Full genome of influenza virus was amplified from the full-length second strand cDNA using forward primer (5′-GCAGATATCAGCRAAAGCAGG-3′) and reverse primer (5′-GCAGATATCAGTAGAAACAAGG-3′), with AccuPrime Taq DNA Polymerase reagents (Thermo Fisher Scientific). A 25 µL PCR reaction was prepared with 2 µL of second strand cDNA according to the manufacturer then incubated with the following cycling conditions: 1 min at 95 °C once, followed by 40 cycles of 30 s at 94 °C, 30 s at 55 °C, and 2 min at 68 °C. PCR products were analyzed with 1% agarose gel electrophoresis and ethidium bromide staining. Then the products were sent to the Centre for Genomic Sciences, HKU for NGS sequencing using Nextera DNA Library preparation and HiSeq 1500 sequencer (Illumina). Sequencing result data were matched to reference influenza virus genomes and call for consensus nucleotides from the aligned reads with at least 100 times sequencing coverage.

### Serological analyses

Serum was extracted from clotted blood samples and stored at −20 °C. The sera were screened using the IDEXX ELISA Influenza A Ab Test (IDEXX Laboratories, Maine, USA), which detects antibody to nucleoprotein of all influenza A viruses^47^ according to the manufacturer’s instructions, with a specimen/negative control (S/N) cut-off value of 0.6. All ELISA positive samples were tested by hemagglutination inhibition (HI) tests. Five viral strains were used as antigen for HI tests. These were three virus strains of different subtype and lineage isolated during the study (human-like H1: A/Swine/Hanoi/826/2014(H1N2); TR H3: A/Swine/Hanoi/415/2013(H3N2); human-like H3: A/Swine/Hanoi/601/2014(H3N2)) together with two other strains (pdm09 H1: A/California/4/2009(H1N1); EA-H1: A/Swine/Hong Kong/4816/2011(H1N1)). The sera were first treated overnight at 37 °C with receptor destroying enzyme (RDE) by mixing one volume of serum with three volumes of RDE previously dissolved in a 0.9% NaCl solution. Next morning, they were heat-inactivated for 30 min at 56 °C and diluted with six volumes of 0.9% NaCl solution. HI tests were performed following the WHO recommendations for animal influenza diagnosis^48^. Briefly, serial twofold dilutions starting with 25 µL of serum in 25 µL of PBS were done in 96-well plates and 25 µL of virus antigen with an HA titer of 8 was added to the equal volume of serum dilution in each well. After 1 h incubation, 50 µL of a 0.5% solution in PBS of washed turkey red blood cells (TRBC) was added to each well and incubated for 30 min. The plates were inspected with tilting. The HI titer of each serum was defined as the highest serum dilution that inhibited hemagglutination and formed a clear button of TRBC at the bottom of the well with a tear drop appearance being seen when the plate was tilted.

### Phylogenetic analyses

The genomic sequences obtained for the virus isolates were analyzed together with other closely related influenza viruses and viruses representing key virus lineages and previously available SIV sequences from Vietnam. The relevant virus sequences were downloaded from GenBank and GISAID. HA and NA sequences from human H1N1pdm09 viruses isolated in 2009-2014 were kindly provided by Dr Le Quynh Mai, National Institute of Hygiene and Epidemiology (NIHE), Hanoi^32^. HA genes of H1N1pdm09, H1N2, and H3N2 viruses, and N1 and N2 genes of viruses were aligned individually by gene subtype using Muscle algorithm in MEGA 6.06^47^. Phylogenetic trees were built using PhyML with approximate likelihood ratio test (aLRT) SH-like branch support^48^. Branch support aLRT statistics were shown at major nodes with values larger or equal to 0.8. For internal genes, sequence alignment was performed using Muscle algorithm as mentioned before. Phylogenetic trees were built in MEGA 6.06 using the Maximum-likelihood method (ML) with 500 bootstrap replications. Bootstrap values greater or equal to 70 were shown on the trees.

### Analysis of surveillance data

The analyses were performed in R 3.2.1^49^ and data were plotted using the ggplot2 package ^50^. Viral isolation rates and HI seroprevalence were calculated for the different locations, over time, and according to the origins of the sampled pigs. Pearson’s chi-squared test with Yates’s correction for continuity was used to investigate potential differences in the virus isolation rates according to farm category of origin. Regarding HI test results, potential cross-reactions between strains of the same HA subtype were analyzed using scatter plots of the HI titers.

For analysis shown in Fig. 6, sera from slaughterhouses were used. When there were sera positive to several strains of the same HA subtype, the antigen with the highest antibody titer (≥4-fold difference between the HI titers) was denoted as the infecting virus; When the difference between antigens was <4-fold, the strain for the subtype was qualified as undetermined (e.g., H1 undetermined).

For each viral strain, a logistic regression was performed using HI seroprevalence data to investigate whether there was a statistical difference of virus circulation in different farming systems and provinces and also to investigate whether the serological results were related to virus isolation on the same month. Only results from slaughterhouses were used (pigs of the same age and from a similar sampling design) and all HI tests results were included, i.e., not taking into account potential cross-reactions between strains of the same HA. The outcome variable was whether a serum was positive or negative by HI test to the strain of interest. The independent variables considered were the sampling location (collective or local slaughterhouse), the farm of origin (familial or company), the province of origin (Hung Yen, Hanoi, or other), whether the strain used as antigen in serological testing had been isolated within the same sampling month (yes/no), and whether the sampling month was before or after the first isolation of the strain of interest. Each independent variable was tested in univariate analysis. Only the variables with *p*-values < 0.2 were then considered for the final model in the multivariate analysis, and in general, the model with the lowest Akaike Information Criterion (AIC) was considered for selection. Also potential correlations between independent variables were checked, and variables considered too correlated were not included together in the model.

## Electronic supplementary material

Supplementary Figure S1

Supplementary Table S2

Supplementary Table S3

Supplementary Figure S4

Supplementary Figure S5

Supplementary Figure S6

Supplementary Figure S7

Supplementary Figure S8

## References

[1] Smith GJ (2009). Origins and evolutionary genomics of the 2009 swine-origin H1N1 influenza A epidemic. Nature.

[2] Mena I (2016). Origins of the 2009 H1N1 influenza pandemic in swine in Mexico. eLife.

[3] Smith GJD (2009). Dating the emergence of pandemic influenza viruses. Proc. Natl Acad. Sci. USA.

[4] Webster RG, Bean WJ, Gorman OT, Chambers TM, Kawaoka Y (1992). Evolution and ecology of influenza A viruses. Microbiol. Rev..

[5] Vijaykrishna D (2011). Long-term evolution and transmission dynamics of swine influenza A virus. Nature.

[6] Vincent A (2014). Review of influenza A virus in swine worldwide: a call for increased surveillance and research. Zoonoses Public Health.

[7] Nelson MI, Vincent AL (2015). Reverse zoonosis of influenza to swine: new perspectives on the human–animal interface. Trends Microbiol..

[8] Shortridge KF, Webster RG, Butterfield WK, Campbell CH (1977). Persistence of Hong Kong influenza virus variants in pigs. Science.

[9] WHO. (2017). Zoonotic influenza viruses: antigenic and genetic characteristics and development of candidate vaccine viruses for pandemic preparedness. Wkly. Epidemiol. Rec..

[10] CDC. Influenza A (H3N2) variant virus: centers for disease control and prevention. http://www.cdc.gov/flu/swineflu/h3n2v-cases.htm. Accessed 1 Dec 2016 (2015).

[11] Coker RJ, Hunter BM, Rudge JW, Liverani M, Hanvoravongchai P (2011). Emerging infectious diseases in southeast Asia: regional challenges to control. Lancet.

[12] Zhu H (2013). History of Swine influenza viruses in Asia. Curr. Top. Microbiol Immunol..

[13] Nelson MI (2015). Global migration of influenza A viruses in swine. Nat. Commun..

[14] WHO. Avian influenza A(H7N9)virus. http://www.who.int/influenza/human_animal_interface/influenza_h7n9/en/. Accessed 30 Nov 2015 (2015).

[15] CDC. Highly pathogenic Asian Avian Influenza A (H5N1) in people: Centers for Disease Control and Prevention. http://www.cdc.gov/flu/avianflu/h5n1-people.htm. Accessed 30 Nov 2015 (2015).

[16] GSO. General Statistics Office of Vietnam. Area, population and population density in 2013 by province. http://www.gso.gov.vn/. Accessed 18 November 2015 (2015).

[17] GSO. General Statistics Office of Vietnam. Number of poultry by province in 2013. http://www.gso.gov.vn/. Accessed 18 November 2015 (2015).

[18] GSO. General Statistics Office of Vietnam. Number of pigs by province in 2013. http://www.gso.gov.vn/. Accessed 17 Nov 2015 (2015).

[19] FAO. *Poultry Production Systems in Vietnam* (FAO, Rome, 2008).

[20] Fisher H, Gordon J (2008). Breeding and feeding pigs in Vietnam: assessment of capacity building and an update on impacts. ACIAR Impact Assess. Ser. Rep..

[21] Delabouglise A (2015). The perceived value of passive animal health surveillance: the case of highly pathogenic avian influenza in Vietnam. Zoonoses Public Health.

[22] Nguyen YT (2013). National surveillance for influenza and influenza-like illness in Vietnam, 2006-2010. Vaccine.

[23] Lemon SM, Hamburg MA, Sparling PF, Choffnes ER, Mack A (2007). Global Infectious Disease Surveillance and Detection: Assessing the Challenges-Finding Solutions, Workshop Summary.

[24] Goutard FL (2015). How to reach the poor? Surveillance in low-income countries, lessons from experiences in Cambodia and Madagascar. Prev. Vet. Med..

[25] Baudon E (2015). Analysis of swine movements in a province in Northern Vietnam and application in the design of surveillance strategies for infectious diseases. Transbound. Emerg. Dis..

[26] Takemae N (2013). Antigenic variation of H1N1, H1N2 and H3N2 swine influenza viruses in Japan and Vietnam. Arch. Virol..

[27] Baudon E (2015). Detection of novel reassortant Influenza A (H3N2) and H1N1 2009 pandemic viruses in swine in Hanoi, Vietnam. Zoonoses Public Health.

[28] Takemae, N. et al. Influenza A viruses of swine (IAV-S) in Vietnam from 2010 to 2015: multiple Introductions of A(H1N1)pdm09 viruses into the pig population and diversifying genetic constellations of enzootic IAV-S. *J. Virol.* **91**, e01490-16 (2017). 10.1128/JVI.01490-16 PMC5165217 27795418

[29] Pascua PN (2013). Emergence of H3N2pM-like and novel reassortant H3N1 swine viruses possessing segments derived from the A (H1N1)pdm09 influenza virus, Korea. Influenza Other Respir. Virus.

[30] Ngo LT (2012). Isolation of novel triple-reassortant swine H3N2 influenza viruses possessing the hemagglutinin and neuraminidase genes of a seasonal influenza virus in Vietnam in 2010. Influenza Other Respir. Virus.

[31] Liang H (2014). Expansion of genotypic diversity and establishment of 2009 H1N1 pandemic-origin internal genes in pigs in China. J. Virol..

[32] Nguyen HK (2015). Virological characterization of influenza H1N1pdm09 in Vietnam, 2010-2013. Influenza Other Respir. Virus.

[33] WHO. World Health Organization. FluNet database. http://www.who.int/influenza/gisrs_laboratory/flunet/en/. Accessed 21 Sept 2015 (2015).

[34] Janke BH (2013). Clinicopathological features of swine influenza. Curr. Top. Microbiol Immunol..

[35] Neira V (2016). Characterization of viral load, viability and persistence of influenza A virus in air and on surfaces of swine production facilities. PLoS ONE.

[36] Van Reeth K, Labarque G, Pensaert M (2006). Serological profiles after consecutive experimental infections of pigs with European H1N1, H3N2, and H1N2 swine influenza viruses. Viral Immunol..

[37] Baudon E, Peyre M, Peiris M, Cowling BJ (2017). Epidemiological features of influenza circulation in swine populations: a systematic review and meta-analysis. PLoS ONE.

[38] Trevennec K (2011). Swine influenza surveillance in East and Southeast Asia: a systematic review. Anim. Health Res Rev..

[39] Takemae, N. et al. Patterns of genetic reassortment between endemic swine influenza viruses and pandemic A(H1N1)2009 viruses in the Vietnamese pig population. In: *Options for the Control of Influenza; Cape Town, South Africa, 5–10 Sep 2013* (2013).

[40] Trevennec K (2012). Transmission of pandemic influenza H1N1 (2009) in Vietnamese swine in 2009-2010. Influenza Other Respir. Virus.

[41] Nelson MI, Stratton J, Killian ML, Janas-Martindale A, Vincent AL (2015). Continual Reintroduction of Human Pandemic H1N1 Influenza A Viruses into Swine in the United States, 2009 to 2014. J. Virol..

[42] Nguyen N. Q. (ed) *Vietnam Pig Systems and R&D Contribution to the Poor. Pig Systems in Asia and the Pacific: How Can Research and Development Enhance Benefits to the Poor?* (ILRI, Bangkok, Thailand, 2006).

[43] Perera HK (2014). Molecular epidemiology of influenza A(H1N1)pdm09 virus among humans and swine, Sri Lanka. Emerg. Infect. Dis..

[44] Ozawa M (2015). Efficient isolation of swine influenza viruses by age-targeted specimen collection. J. Clin. Microbiol..

[45] Mak PW (2011). Rapid genotyping of swine influenza viruses. Emerg. Infect. Dis..

[46] Hoffmann E, Stech J, Guan Y, Webster RG, Perez DR (2001). Universal primer set for the full-length amplification of all influenza A viruses. Arch. Virol..

[47] Tse M (2012). Evaluation of three commercially available influenza A type-specific blocking enzyme-linked immunosorbent assays for seroepidemiological studies of influenza A virus infection in pigs. Clin. Vaccin. Immunol..

[48] Webster, R. G., Cox, N., & Stohr, K. WHO manual on animal influenza diagnosis and surveillance. World Health Organization, Department of Communicable Disease Surveillance and Response. WHO/CDS/CDR/2002.5 Rev. 1. http://www.who.int/csr/resources/publications/influenza/en/whocdscsrncs20025rev.pdf. Accessed April 2013 (2002).

[49] R Core Team. (2015). R: A Language and Environment for Statistical Computing.

[50] Wickham, H. *ggplot2: Elegant Graphics for Data Analysis* (Springer, New York, 2009).

