# Supplementary material for: Swine influenza viruses in Northern Vietnam in 2013–2014
Source: Emerg Microbes Infect. 2018 Jul 2;7:123. doi: 10.1038/s41426-018-0109-y (PMC6028489; doi:10.1038/s41426-018-0109-y)
Supplement: Supplementary file 1 — Supplementary Figure S1 [file 41426_2018_109_MOESM1_ESM.pdf]

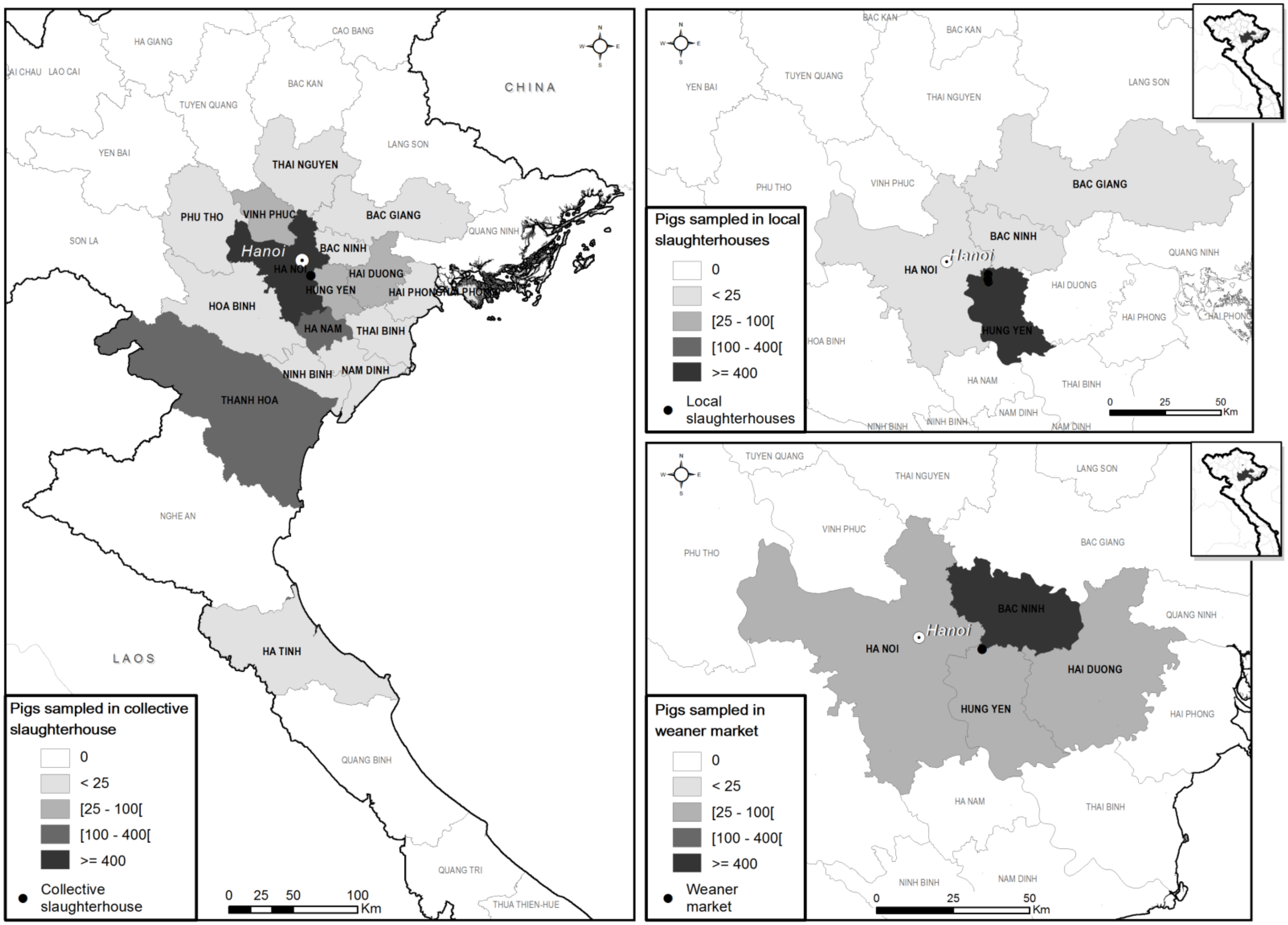

Supplementary Figure S1. Provinces of origin and number of pigs per province for the pigs sampled in the different pilot surveillance protocols from 2013 to 2014 in Hung Yen and Hanoi
