# Supplementary material for: Swine influenza viruses in Northern Vietnam in 2013–2014
Source: Emerg Microbes Infect. 2018 Jul 2;7:123. doi: 10.1038/s41426-018-0109-y (PMC6028489; doi:10.1038/s41426-018-0109-y)
Supplement: Supplementary file 2 — Supplementary Table S2 [file 41426_2018_109_MOESM2_ESM.pdf]

Supplementary Table S2. List of viruses isolated in the collective slaughterhouse in Hanoi in 2013-2014

Isolation date	Subtype	Strain name
**H1N1**		
Aug-13	H1N1	**A/swine/Hanoi/411/2013(H1N1)**
Aug-13	H1N1	**A/swine/Hanoi/424/2013(H1N1)**
Aug-13	H1N1	A/swine/Hanoi/443/2013(H1N1)
Aug-13	H1N1	A/swine/Hanoi/445/2013(H1N1)
Sep-13	H1N1	A/swine/Hanoi/568/2013(H1N1)
Sep-13	H1N1	A/swine/Hanoi/571/2013(H1N1)
Oct-13	H1N1	A/swine/Hanoi/834/2013(H1N1)
Oct-13	H1N1	A/swine/Hanoi/836/2013(H1N1)
Nov-13	H1N1	**A/swine/Hanoi/934/2013(H1N1)**
Nov-13	H1N1	**A/swine/Hanoi/952/2013(H1N1)**
Mar-14	H1N1	**A/swine/Hanoi/586/2014(H1N1)**
Mar-14	H1N1	**A/swine/Hanoi/588/2014(H1N1)**
May-14	H1N1	**A/swine/Hanoi/788/2014(H1N1)**
May-14	H1N1	**A/swine/Hanoi/838/2014(H1N1)**
May-14	H1N1	**A/swine/Hanoi/872/2014(H1N1)**
May-14	H1N1	**A/swine/Hanoi/894/2014(H1N1)**
**H1N2**		
Dec-13	H1N2	**A/swine/Hanoi/054/2013(H1N2)**
Jan-14	H1N2	**A/swine/Hanoi/304/2014(H1N2)**
Mar-14	H1N2	**A/swine/Hanoi/532/2014(H1N2)**
Mar-14	H1N2	A/swine/Hanoi/534/2014(H1N2)
Mar-14	H1N2	A/swine/Hanoi/541/2014(H1N2)
Mar-14	H1N2	**A/swine/Hanoi/542/2014(H1N2)**
Mar-14	H1N2	A/swine/Hanoi/544/2014(H1N2)
Mar-14	H1N2	**A/swine/Hanoi/546/2014(H1N2)**
Mar-14	H1N2	**A/swine/Hanoi/547/2014(H1N2)**
Mar-14	H1N2	A/swine/Hanoi/554/2014(H1N2)
Mar-14	H1N2	**A/swine/Hanoi/556/2014(H1N2)**

Isolation date	Subtype	Strain name
**H1N2 (Cont.)**		
Apr-14	H1N2	**A/swine/Hanoi/643/2014(H1N2)**
May-14	H1N2	**A/swine/Hanoi/815/2014(H1N2)**
May-14	H1N2	A/swine/Hanoi/817/2014(H1N2)
May-14	H1N2	**A/swine/Hanoi/819/2014(H1N2)**
May-14	H1N2	A/swine/Hanoi/820/2014(H1N2)
May-14	H1N2	**A/swine/Hanoi/822/2014(H1N2)**
May-14	H1N2	**A/swine/Hanoi/824/2014(H1N2)**
May-14	H1N2	**A/swine/Hanoi/825/2014(H1N2)**
May-14	H1N2	**A/swine/Hanoi/826/2014(H1N2)**
May-14	H1N2	**A/swine/Hanoi/830/2014(H1N2)**
May-14	H1N2	A/swine/Hanoi/831/2014(H1N2)
May-14	H1N2	**A/swine/Hanoi/833/2014(H1N2)**
May-14	H1N2	**A/swine/Hanoi/836/2014(H1N2)**
May-14	H1N2	A/swine/Hanoi/839/2014(H1N2)
May-14	H1N2	**A/swine/Hanoi/888/2014(H1N2)**
May-14	H1N2	**A/swine/Hanoi/896/2014(H1N2)**
May-14	H1N2	A/swine/Hanoi/897/2014(H1N2)
May-14	H1N2	A/swine/Hanoi/898/2014(H1N2)
May-14	H1N2	**A/swine/Hanoi/900/2014(H1N2)**
May-14	H1N2	**A/swine/Hanoi/907/2014(H1N2)**
May-14	H1N2	A/swine/Hanoi/908/2014(H1N2)
May-14	H1N2	A/swine/Hanoi/910/2014(H1N2)
May-14	H1N2	A/swine/Hanoi/911/2014(H1N2)
May-14	H1N2	A/swine/Hanoi/912/2014(H1N2)

Legend:
A/swine: Full length sequencing for all eight segments
A/swine: Partial sequencing only for internal genes

Supplementary Table S2. List of viruses isolated in the collective slaughterhouse in Hanoi in 2013-2014 (Continued)

Isolation date	Subtype	Strain name
**H3N2 related to Korean TR**		
Aug-13	H3N2	A/swine/Hanoi/415/2013(H3N2)
Aug-13	H3N2	A/swine/Hanoi/417/2013(H3N2)
Aug-13	H3N2	A/swine/Hanoi/420/2013(H3N2)
Aug-13	H3N2	A/swine/Hanoi/421/2013(H3N2)
Aug-13	H3N2	A/swine/Hanoi/422/2013(H3N2)
Aug-13	H3N2	A/swine/Hanoi/435/2013(H3N2)
Aug-13	H3N2	A/swine/Hanoi/437/2013(H3N2)
Aug-13	H3N2	A/swine/Hanoi/447/2013(H3N2)
Aug-13	H3N2	A/swine/Hanoi/454/2013(H3N2)
Dec-13	H3N2	**A/swine/Hanoi/027/2013(H3N2)**
Jan-14	H3N2	**A/swine/Hanoi/181/2014(H3N2)**
Jan-14	H3N2	A/swine/Hanoi/190/2014(H3N2)
Jan-14	H3N2	**A/swine/Hanoi/192/2014(H3N2)**
Feb-14	H3N2	**A/swine/Hanoi/351/2014(H3N2)**
Feb-14	H3N2	**A/swine/Hanoi/353/2014(H3N2)**
Feb-14	H3N2	A/swine/Hanoi/355/2014(H3N2)
Feb-14	H3N2	**A/swine/Hanoi/359/2014(H3N2)**
Feb-14	H3N2	A/swine/Hanoi/361/2014(H3N2)
Feb-14	H3N2	**A/swine/Hanoi/405/2014(H3N2)**
Feb-14	H3N2	**A/swine/Hanoi/434/2014(H3N2)**
Feb-14	H3N2	**A/swine/Hanoi/442/2014(H3N2)**
Feb-14	H3N2	A/swine/Hanoi/444/2014(H3N2)
Apr-14	H3N2	**A/swine/Hanoi/647/2014(H3N2)**
Apr-14	H3N2	A/swine/Hanoi/649/2014(H3N2)
Apr-14	H3N2	**A/swine/Hanoi/655/2014(H3N2)**
**H3N2 Binh Duong-like**		
Mar-14	H3N2 BD-like	**A/swine/Hanoi/601/2014(H3N2)**
