# Supplementary material for: Swine influenza viruses in Northern Vietnam in 2013–2014
Source: Emerg Microbes Infect. 2018 Jul 2;7:123. doi: 10.1038/s41426-018-0109-y (PMC6028489; doi:10.1038/s41426-018-0109-y)
Supplement: Supplementary file 3 — Supplementary Table S3 [file 41426_2018_109_MOESM3_ESM.pdf]

Supplementary Table S3. Number of influenza viruses isolated and isolation rate per month at the collective slaughterhouse

Date of sampling	Number of nasal swab samples	Number of isolates	Isolation rate %	Subtypes	Genotypes*
07-May-13	121	0	0	-	-
11-Jun-13	130	0	0	-	-
02-Jul-13	150	0	0	-	-
06-Aug-13	150	13	8.7	H1N1, H3N2-K	1, 14
04-Sep-13	150	2	1.3	H1N1	1
02-Oct-13	150	2	1.3	H1N1	-
06-Nov-13	150	2	1.3	H1N1	1
04-Dec-13	150	2	1.3	H1N2, H3N2-K	8, 14
17-Jan-14	150	4	2.7	H1N2, H3N2-K	8, 16
13-Feb-14	150	9	6.0	H3N2-K	14, 15
19-Mar-14	150	12	8.0	H1N1, H1N2, H3N2-BD	1, 8, 12
02-Apr-14	150	4	2.7	H1N2, H3N2-K	8, 16
08-May-14	150	27	18	H1N1, H1N2	1, 8
11-Jun-14	150	0	0	-	-
**Total**	**2,051**	**77**	**3.8**		

*Genotypes of the sequenced viruses
H3N2-K=Korean-like H3N2 ; H3N2-BD=Binh Duong-like H3N2
