# Supplementary material for: Swine influenza viruses in Northern Vietnam in 2013–2014
Source: Emerg Microbes Infect. 2018 Jul 2;7:123. doi: 10.1038/s41426-018-0109-y (PMC6028489; doi:10.1038/s41426-018-0109-y)
Supplement: Supplementary file 4 — Supplementary Figure S4 [file 41426_2018_109_MOESM4_ESM.pdf]

Supplementary Figure S4. Phylogenetic tree for the full length PB2 of viruses isolated in a slaughterhouse in Vietnam in 2013-2014

The trees were constructed with MEGA 6.06. Bootstrap values greater or equal to 70 are shown. GenBank accession numbers of retrieved sequences are indicated.

Underlined: Viruses from our study. **Bold: Viruses from Vietnam from other studies.**

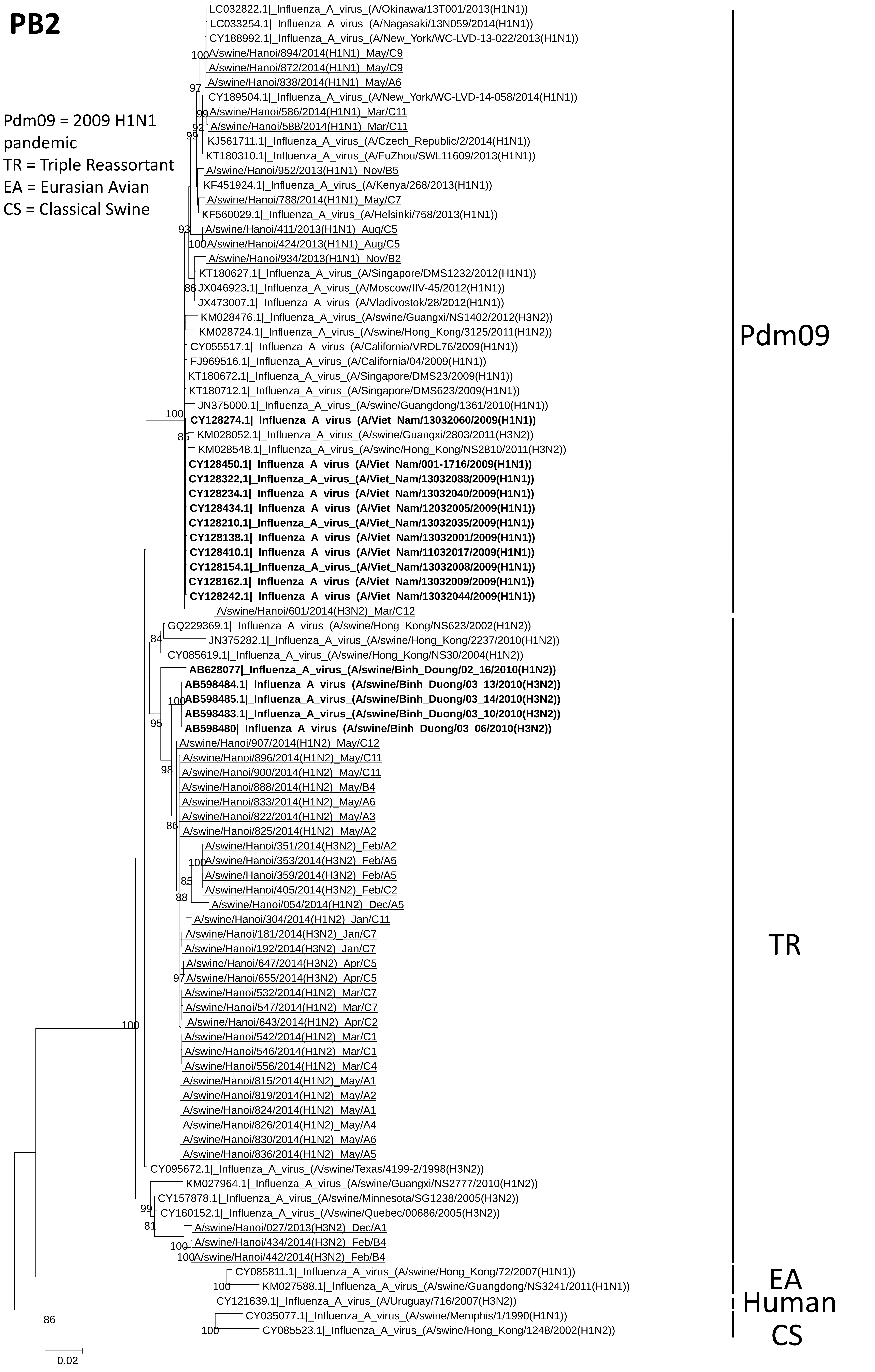

PB2
Pdm09 = 2009 H1N1 pandemic
TR = Triple Reassortant
EA = Eurasian Avian
CS = Classical Swine
LC032822.1|_Influenza_A_virus_(A/Okinawa/13T001/2013(H1N1))
LC033254.1|_Influenza_A_virus_(A/Nagasaki/13N059/2014(H1N1))
CY188992.1|_Influenza_A_virus_(A/New_York/WC-LVD-13-022/2013(H1N1))
A/swine/Hanoi/894/2014(H1N1)_May/C9
A/swine/Hanoi/872/2014(H1N1)_May/C9
A/swine/Hanoi/838/2014(H1N1)_May/A6
CY189504.1|_Influenza_A_virus_(A/New_York/WC-LVD-14-058/2014(H1N1))
A/swine/Hanoi/586/2014(H1N1)_Mar/C11
A/swine/Hanoi/588/2014(H1N1)_Mar/C11
KJ561711.1|_Influenza_A_virus_(A/Czech_Republic/2/2014(H1N1))
KT180310.1|_Influenza_A_virus_(A/FuZhou/SWL11609/2013(H1N1))
A/swine/Hanoi/952/2013(H1N1)_Nov/B5
KF451924.1|_Influenza_A_virus_(A/Kenya/268/2013(H1N1))
A/swine/Hanoi/788/2014(H1N1)_May/C7
KF560029.1|_Influenza_A_virus_(A/Helsinki/758/2013(H1N1))
A/swine/Hanoi/411/2013(H1N1)_Aug/C5
A/swine/Hanoi/424/2013(H1N1)_Aug/C5
A/swine/Hanoi/934/2013(H1N1)_Nov/B2
KT180627.1|_Influenza_A_virus_(A/Singapore/DMS1232/2012(H1N1))
JX046923.1|_Influenza_A_virus_(A/Moscow/IIV-45/2012(H1N1))
JX473007.1|_Influenza_A_virus_(A/Vladivostok/28/2012(H1N1))
KM028476.1|_Influenza_A_virus_(A/swine/Guangxi/NS1402/2012(H3N2))
KM028724.1|_Influenza_A_virus_(A/swine/Hong_Kong/3125/2011(H1N2))
CY055517.1|_Influenza_A_virus_(A/California/VRDL76/2009(H1N1))
FJ969516.1|_Influenza_A_virus_(A/California/04/2009(H1N1))
KT180672.1|_Influenza_A_virus_(A/Singapore/DMS23/2009(H1N1))
KT180712.1|_Influenza_A_virus_(A/Singapore/DMS623/2009(H1N1))
JN375000.1|_Influenza_A_virus_(A/swine/Guangdong/1361/2010(H1N1))
CY128274.1|_Influenza_A_virus_(A/Viet_Nam/13032060/2009(H1N1))
KM028052.1|_Influenza_A_virus_(A/swine/Guangxi/2803/2011(H3N2))
KM028548.1|_Influenza_A_virus_(A/swine/Hong_Kong/NS2810/2011(H3N2))
CY128450.1|_Influenza_A_virus_(A/Viet_Nam/001-1716/2009(H1N1))
CY128322.1|_Influenza_A_virus_(A/Viet_Nam/13032088/2009(H1N1))
CY128234.1|_Influenza_A_virus_(A/Viet_Nam/13032040/2009(H1N1))
CY128434.1|_Influenza_A_virus_(A/Viet_Nam/12032005/2009(H1N1))
CY128210.1|_Influenza_A_virus_(A/Viet_Nam/13032035/2009(H1N1))
CY128138.1|_Influenza_A_virus_(A/Viet_Nam/13032001/2009(H1N1))
CY128410.1|_Influenza_A_virus_(A/Viet_Nam/11032017/2009(H1N1))
CY128154.1|_Influenza_A_virus_(A/Viet_Nam/13032008/2009(H1N1))
CY128162.1|_Influenza_A_virus_(A/Viet_Nam/13032009/2009(H1N1))
CY128242.1|_Influenza_A_virus_(A/Viet_Nam/13032044/2009(H1N1))
A/swine/Hanoi/601/2014(H3N2)_Mar/C12
Pdm09
GQ229369.1|_Influenza_A_virus_(A/swine/Hong_Kong/NS623/2002(H1N2))
JN375282.1|_Influenza_A_virus_(A/swine/Hong_Kong/2237/2010(H1N2))
CY085619.1|_Influenza_A_virus_(A/swine/Hong_Kong/NS30/2004(H1N2))
AB628077|_Influenza_A_virus_(A/swine/Binh_Doung/02_16/2010(H1N2))
AB598484.1|_Influenza_A_virus_(A/swine/Binh_Duong/03_13/2010(H3N2))
AB598485.1|_Influenza_A_virus_(A/swine/Binh_Duong/03_14/2010(H3N2))
AB598483.1|_Influenza_A_virus_(A/swine/Binh_Duong/03_10/2010(H3N2))
AB598480|_Influenza_A_virus_(A/swine/Binh_Duong/03_06/2010(H3N2))
A/swine/Hanoi/907/2014(H1N2)_May/C12
A/swine/Hanoi/896/2014(H1N2)_May/C11
A/swine/Hanoi/900/2014(H1N2)_May/C11
A/swine/Hanoi/888/2014(H1N2)_May/B4
A/swine/Hanoi/833/2014(H1N2)_May/A6
A/swine/Hanoi/822/2014(H1N2)_May/A3
A/swine/Hanoi/825/2014(H1N2)_May/A2
A/swine/Hanoi/351/2014(H3N2)_Feb/A2
A/swine/Hanoi/353/2014(H3N2)_Feb/A5
A/swine/Hanoi/359/2014(H3N2)_Feb/A5
A/swine/Hanoi/405/2014(H3N2)_Feb/C2
A/swine/Hanoi/054/2014(H1N2)_Dec/A5
A/swine/Hanoi/304/2014(H1N2)_Jan/C11
A/swine/Hanoi/181/2014(H3N2)_Jan/C7
A/swine/Hanoi/192/2014(H3N2)_Jan/C7
A/swine/Hanoi/647/2014(H3N2)_Apr/C5
A/swine/Hanoi/655/2014(H3N2)_Apr/C5
A/swine/Hanoi/532/2014(H1N2)_Mar/C7
A/swine/Hanoi/547/2014(H1N2)_Mar/C7
A/swine/Hanoi/643/2014(H1N2)_Apr/C2
A/swine/Hanoi/542/2014(H1N2)_Mar/C1
A/swine/Hanoi/546/2014(H1N2)_Mar/C1
A/swine/Hanoi/556/2014(H1N2)_Mar/C4
A/swine/Hanoi/815/2014(H1N2)_May/A1
A/swine/Hanoi/819/2014(H1N2)_May/A2
A/swine/Hanoi/824/2014(H1N2)_May/A1
A/swine/Hanoi/826/2014(H1N2)_May/A4
A/swine/Hanoi/830/2014(H1N2)_May/A6
A/swine/Hanoi/836/2014(H1N2)_May/A5
CY095672.1|_Influenza_A_virus_(A/swine/Texas/4199-2/1998(H3N2))
KM027964.1|_Influenza_A_virus_(A/swine/Guangxi/NS2777/2010(H1N2))
CY157878.1|_Influenza_A_virus_(A/swine/Minnesota/SG1238/2005(H3N2))
CY160152.1|_Influenza_A_virus_(A/swine/Quebec/00686/2005(H3N2))
A/swine/Hanoi/027/2013(H3N2)_Dec/A1
A/swine/Hanoi/434/2014(H3N2)_Feb/B4
A/swine/Hanoi/442/2014(H3N2)_Feb/B4
TR
CY085811.1|_Influenza_A_virus_(A/swine/Hong_Kong/72/2007(H1N1))
KM027588.1|_Influenza_A_virus_(A/swine/Guangdong/NS3241/2011(H1N1))
EA
CY121639.1|_Influenza_A_virus_(A/Uruguay/716/2007(H3N2))
Human
CY035077.1|_Influenza_A_virus_(A/swine/Memphis/1/1990(H1N1))
CY085523.1|_Influenza_A_virus_(A/swine/Hong_Kong/1248/2002(H1N2))
CS
0.02
