# Supplementary material for: Swine influenza viruses in Northern Vietnam in 2013–2014
Source: Emerg Microbes Infect. 2018 Jul 2;7:123. doi: 10.1038/s41426-018-0109-y (PMC6028489; doi:10.1038/s41426-018-0109-y)
Supplement: Supplementary file 5 — Supplementary Figure S5 [file 41426_2018_109_MOESM5_ESM.pdf]

Supplementary Figure S5. Phylogenetic tree for the full length NP of viruses isolated in a slaughterhouse in Vietnam in 2013-2014

The trees were constructed with MEGA 6.06. Bootstrap values greater or equal to 70 are shown. GenBank accession numbers of retrieved sequences are indicated.

Underlined: Viruses from our study. **Bold: Viruses from Vietnam from other studies.**

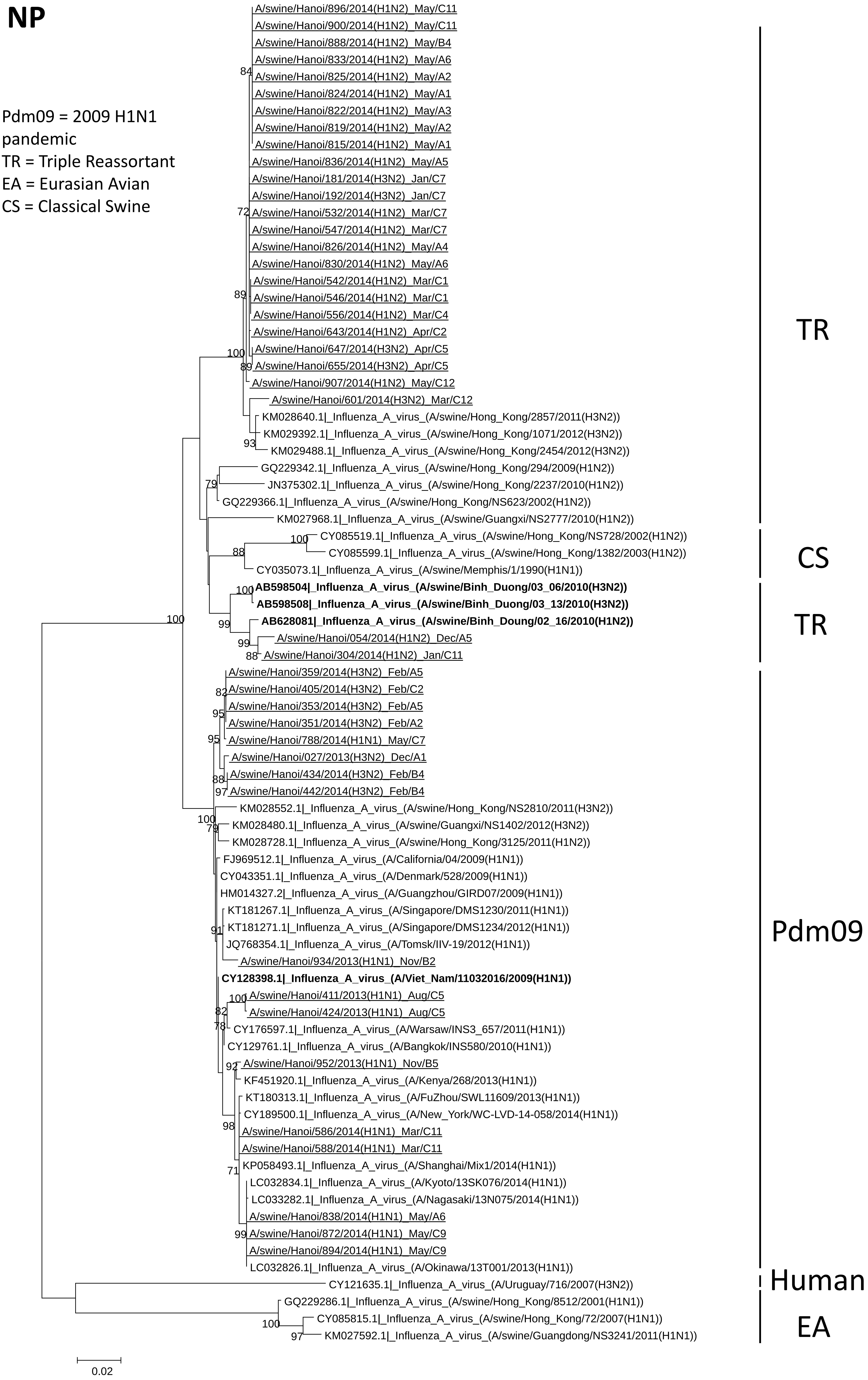

NP
Pdm09 = 2009 H1N1 pandemic
TR = Triple Reassortant
EA = Eurasian Avian
CS = Classical Swine
A/swine/Hanoi/896/2014(H1N2)_May/C11
A/swine/Hanoi/900/2014(H1N2)_May/C11
A/swine/Hanoi/888/2014(H1N2)_May/B4
A/swine/Hanoi/833/2014(H1N2)_May/A6
A/swine/Hanoi/825/2014(H1N2)_May/A2
A/swine/Hanoi/824/2014(H1N2)_May/A1
A/swine/Hanoi/822/2014(H1N2)_May/A3
A/swine/Hanoi/819/2014(H1N2)_May/A2
A/swine/Hanoi/815/2014(H1N2)_May/A1
A/swine/Hanoi/836/2014(H1N2)_May/A5
A/swine/Hanoi/181/2014(H3N2)_Jan/C7
A/swine/Hanoi/192/2014(H3N2)_Jan/C7
A/swine/Hanoi/532/2014(H1N2)_Mar/C7
A/swine/Hanoi/547/2014(H1N2)_Mar/C7
A/swine/Hanoi/826/2014(H1N2)_May/A4
A/swine/Hanoi/830/2014(H1N2)_May/A6
A/swine/Hanoi/542/2014(H1N2)_Mar/C1
A/swine/Hanoi/546/2014(H1N2)_Mar/C1
A/swine/Hanoi/556/2014(H1N2)_Mar/C4
A/swine/Hanoi/643/2014(H1N2)_Apr/C2
A/swine/Hanoi/647/2014(H3N2)_Apr/C5
A/swine/Hanoi/655/2014(H3N2)_Apr/C5
A/swine/Hanoi/907/2014(H1N2)_May/C12
A/swine/Hanoi/601/2014(H3N2)_Mar/C12
KM028640.1|_Influenza_A_virus_(A/swine/Hong_Kong/2857/2011(H3N2))
KM029392.1|_Influenza_A_virus_(A/swine/Hong_Kong/1071/2012(H3N2))
KM029488.1|_Influenza_A_virus_(A/swine/Hong_Kong/2454/2012(H3N2))
GQ229342.1|_Influenza_A_virus_(A/swine/Hong_Kong/294/2009(H1N2))
JN375302.1|_Influenza_A_virus_(A/swine/Hong_Kong/2237/2010(H1N2))
GQ229366.1|_Influenza_A_virus_(A/swine/Hong_Kong/NS623/2002(H1N2))
KM027968.1|_Influenza_A_virus_(A/swine/Guangxi/NS2777/2010(H1N2))
CY085519.1|_Influenza_A_virus_(A/swine/Hong_Kong/NS728/2002(H1N2))
CY085599.1|_Influenza_A_virus_(A/swine/Hong_Kong/1382/2003(H1N2))
CY035073.1|_Influenza_A_virus_(A/swine/Memphis/1/1990(H1N1))
AB598504|_Influenza_A_virus_(A/swine/Binh_Duong/03_06/2010(H3N2))
AB598508|_Influenza_A_virus_(A/swine/Binh_Duong/03_13/2010(H3N2))
AB628081|_Influenza_A_virus_(A/swine/Binh_Doung/02_16/2010(H1N2))
A/swine/Hanoi/054/2014(H1N2)_Dec/A5
A/swine/Hanoi/304/2014(H1N2)_Jan/C11
A/swine/Hanoi/359/2014(H3N2)_Feb/A5
A/swine/Hanoi/405/2014(H3N2)_Feb/C2
A/swine/Hanoi/353/2014(H3N2)_Feb/A5
A/swine/Hanoi/351/2014(H3N2)_Feb/A2
A/swine/Hanoi/788/2014(H1N1)_May/C7
A/swine/Hanoi/027/2013(H3N2)_Dec/A1
A/swine/Hanoi/434/2014(H3N2)_Feb/B4
A/swine/Hanoi/442/2014(H3N2)_Feb/B4
KM028552.1|_Influenza_A_virus_(A/swine/Hong_Kong/NS2810/2011(H3N2))
KM028480.1|_Influenza_A_virus_(A/swine/Guangxi/NS1402/2012(H3N2))
KM028728.1|_Influenza_A_virus_(A/swine/Hong_Kong/3125/2011(H1N2))
FJ969512.1|_Influenza_A_virus_(A/California/04/2009(H1N1))
CY043351.1|_Influenza_A_virus_(A/Denmark/528/2009(H1N1))
HM014327.2|_Influenza_A_virus_(A/Guangzhou/GIRD07/2009(H1N1))
KT181267.1|_Influenza_A_virus_(A/Singapore/DMS1230/2011(H1N1))
KT181271.1|_Influenza_A_virus_(A/Singapore/DMS1234/2012(H1N1))
JQ768354.1|_Influenza_A_virus_(A/Tomsk/IIV-19/2012(H1N1))
A/swine/Hanoi/934/2013(H1N1)_Nov/B2
CY128398.1|_Influenza_A_virus_(A/Viet_Nam/11032016/2009(H1N1))
A/swine/Hanoi/411/2013(H1N1)_Aug/C5
A/swine/Hanoi/424/2013(H1N1)_Aug/C5
CY176597.1|_Influenza_A_virus_(A/Warsaw/INS3_657/2011(H1N1))
CY129761.1|_Influenza_A_virus_(A/Bangkok/INS580/2010(H1N1))
A/swine/Hanoi/952/2013(H1N1)_Nov/B5
KF451920.1|_Influenza_A_virus_(A/Kenya/268/2013(H1N1))
KT180313.1|_Influenza_A_virus_(A/FuZhou/SWL11609/2013(H1N1))
CY189500.1|_Influenza_A_virus_(A/New_York/WC-LVD-14-058/2014(H1N1))
A/swine/Hanoi/586/2014(H1N1)_Mar/C11
A/swine/Hanoi/588/2014(H1N1)_Mar/C11
KP058493.1|_Influenza_A_virus_(A/Shanghai/Mix1/2014(H1N1))
LC032834.1|_Influenza_A_virus_(A/Kyoto/13SK076/2014(H1N1))
LC033282.1|_Influenza_A_virus_(A/Nagasaki/13N075/2014(H1N1))
A/swine/Hanoi/838/2014(H1N1)_May/A6
A/swine/Hanoi/872/2014(H1N1)_May/C9
A/swine/Hanoi/894/2014(H1N1)_May/C9
LC032826.1|_Influenza_A_virus_(A/Okinawa/13T001/2013(H1N1))
CY121635.1|_Influenza_A_virus_(A/Uruguay/716/2007(H3N2))
GQ229286.1|_Influenza_A_virus_(A/swine/Hong_Kong/8512/2001(H1N1))
CY085815.1|_Influenza_A_virus_(A/swine/Hong_Kong/72/2007(H1N1))
KM027592.1|_Influenza_A_virus_(A/swine/Guangdong/NS3241/2011(H1N1))
TR
CS
TR
Pdm09
Human
EA
0.02
