# Supplementary material for: Swine influenza viruses in Northern Vietnam in 2013–2014
Source: Emerg Microbes Infect. 2018 Jul 2;7:123. doi: 10.1038/s41426-018-0109-y (PMC6028489; doi:10.1038/s41426-018-0109-y)
Supplement: Supplementary file 6 — Supplementary Figure S6 [file 41426_2018_109_MOESM6_ESM.pdf]

Supplementary Figure S6. Phylogenetic tree for the full length M of viruses isolated in a slaughterhouse in Vietnam in 2013-2014

The trees were constructed with MEGA 6.06. Bootstrap values greater or equal to 70 are shown. GenBank accession numbers of retrieved sequences are indicated.

Underlined: Viruses from our study. **Bold: Viruses from Vietnam from other studies.**

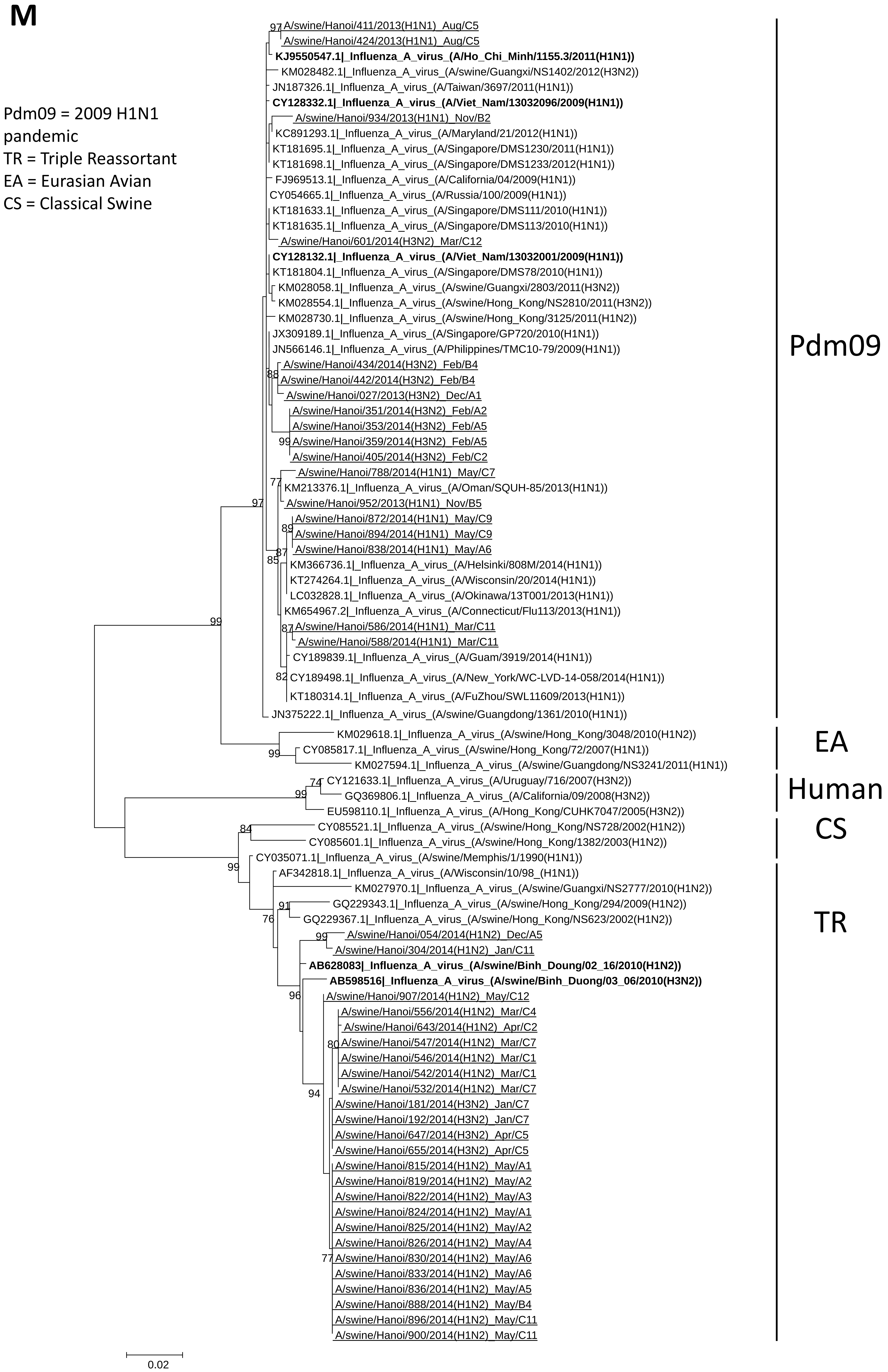
M
Pdm09 = 2009 H1N1 pandemic
TR = Triple Reassortant
EA = Eurasian Avian
CS = Classical Swine
97
A/swine/Hanoi/411/2013(H1N1)_Aug/C5
A/swine/Hanoi/424/2013(H1N1)_Aug/C5
KJ9550547.1|_Influenza_A_virus_(A/Ho_Chi_Minh/1155.3/2011(H1N1))
KM028482.1|_Influenza_A_virus_(A/swine/Guangxi/NS1402/2012(H3N2))
JN187326.1|_Influenza_A_virus_(A/Taiwan/3697/2011(H1N1))
CY128332.1|_Influenza_A_virus_(A/Viet_Nam/13032096/2009(H1N1))
A/swine/Hanoi/934/2013(H1N1)_Nov/B2
KC891293.1|_Influenza_A_virus_(A/Maryland/21/2012(H1N1))
KT181695.1|_Influenza_A_virus_(A/Singapore/DMS1230/2011(H1N1))
KT181698.1|_Influenza_A_virus_(A/Singapore/DMS1233/2012(H1N1))
FJ969513.1|_Influenza_A_virus_(A/California/04/2009(H1N1))
CY054665.1|_Influenza_A_virus_(A/Russia/100/2009(H1N1))
KT181633.1|_Influenza_A_virus_(A/Singapore/DMS111/2010(H1N1))
KT181635.1|_Influenza_A_virus_(A/Singapore/DMS113/2010(H1N1))
A/swine/Hanoi/601/2014(H3N2)_Mar/C12
CY128132.1|_Influenza_A_virus_(A/Viet_Nam/13032001/2009(H1N1))
KT181804.1|_Influenza_A_virus_(A/Singapore/DMS78/2010(H1N1))
KM028058.1|_Influenza_A_virus_(A/swine/Guangxi/2803/2011(H3N2))
KM028554.1|_Influenza_A_virus_(A/swine/Hong_Kong/NS2810/2011(H3N2))
KM028730.1|_Influenza_A_virus_(A/swine/Hong_Kong/3125/2011(H1N2))
JX309189.1|_Influenza_A_virus_(A/Singapore/GP720/2010(H1N1))
JN566146.1|_Influenza_A_virus_(A/Philippines/TMC10-79/2009(H1N1))
A/swine/Hanoi/434/2014(H3N2)_Feb/B4
88
A/swine/Hanoi/442/2014(H3N2)_Feb/B4
A/swine/Hanoi/027/2013(H3N2)_Dec/A1
A/swine/Hanoi/351/2014(H3N2)_Feb/A2
A/swine/Hanoi/353/2014(H3N2)_Feb/A5
99
A/swine/Hanoi/359/2014(H3N2)_Feb/A5
A/swine/Hanoi/405/2014(H3N2)_Feb/C2
A/swine/Hanoi/788/2014(H1N1)_May/C7
77
KM213376.1|_Influenza_A_virus_(A/Oman/SQUH-85/2013(H1N1))
97
A/swine/Hanoi/952/2013(H1N1)_Nov/B5
A/swine/Hanoi/872/2014(H1N1)_May/C9
89
A/swine/Hanoi/894/2014(H1N1)_May/C9
A/swine/Hanoi/838/2014(H1N1)_May/A6
87
85
KM366736.1|_Influenza_A_virus_(A/Helsinki/808M/2014(H1N1))
KT274264.1|_Influenza_A_virus_(A/Wisconsin/20/2014(H1N1))
LC032828.1|_Influenza_A_virus_(A/Okinawa/13T001/2013(H1N1))
KM654967.2|_Influenza_A_virus_(A/Connecticut/Flu113/2013(H1N1))
99
87
A/swine/Hanoi/586/2014(H1N1)_Mar/C11
A/swine/Hanoi/588/2014(H1N1)_Mar/C11
CY189839.1|_Influenza_A_virus_(A/Guam/3919/2014(H1N1))
82
CY189498.1|_Influenza_A_virus_(A/New_York/WC-LVD-14-058/2014(H1N1))
KT180314.1|_Influenza_A_virus_(A/FuZhou/SWL11609/2013(H1N1))
JN375222.1|_Influenza_A_virus_(A/swine/Guangdong/1361/2010(H1N1))
Pdm09
KM029618.1|_Influenza_A_virus_(A/swine/Hong_Kong/3048/2010(H1N2))
99
CY085817.1|_Influenza_A_virus_(A/swine/Hong_Kong/72/2007(H1N1))
KM027594.1|_Influenza_A_virus_(A/swine/Guangdong/NS3241/2011(H1N1))
EA
74
CY121633.1|_Influenza_A_virus_(A/Uruguay/716/2007(H3N2))
99
GQ369806.1|_Influenza_A_virus_(A/California/09/2008(H3N2))
EU598110.1|_Influenza_A_virus_(A/Hong_Kong/CUHK7047/2005(H3N2))
Human
84
CY085521.1|_Influenza_A_virus_(A/swine/Hong_Kong/NS728/2002(H1N2))
CY085601.1|_Influenza_A_virus_(A/swine/Hong_Kong/1382/2003(H1N2))
CS
CY035071.1|_Influenza_A_virus_(A/swine/Memphis/1/1990(H1N1))
99
AF342818.1|_Influenza_A_virus_(A/Wisconsin/10/98_(H1N1))
KM027970.1|_Influenza_A_virus_(A/swine/Guangxi/NS2777/2010(H1N2))
91
GQ229343.1|_Influenza_A_virus_(A/swine/Hong_Kong/294/2009(H1N2))
76
GQ229367.1|_Influenza_A_virus_(A/swine/Hong_Kong/NS623/2002(H1N2))
99
A/swine/Hanoi/054/2014(H1N2)_Dec/A5
A/swine/Hanoi/304/2014(H1N2)_Jan/C11
AB628083|_Influenza_A_virus_(A/swine/Binh_Doung/02_16/2010(H1N2))
AB598516|_Influenza_A_virus_(A/swine/Binh_Duong/03_06/2010(H3N2))
96
A/swine/Hanoi/907/2014(H1N2)_May/C12
A/swine/Hanoi/556/2014(H1N2)_Mar/C4
A/swine/Hanoi/643/2014(H1N2)_Apr/C2
80
A/swine/Hanoi/547/2014(H1N2)_Mar/C7
A/swine/Hanoi/546/2014(H1N2)_Mar/C1
A/swine/Hanoi/542/2014(H1N2)_Mar/C1
A/swine/Hanoi/532/2014(H1N2)_Mar/C7
94
A/swine/Hanoi/181/2014(H3N2)_Jan/C7
A/swine/Hanoi/192/2014(H3N2)_Jan/C7
A/swine/Hanoi/647/2014(H3N2)_Apr/C5
A/swine/Hanoi/655/2014(H3N2)_Apr/C5
A/swine/Hanoi/815/2014(H1N2)_May/A1
A/swine/Hanoi/819/2014(H1N2)_May/A2
A/swine/Hanoi/822/2014(H1N2)_May/A3
A/swine/Hanoi/824/2014(H1N2)_May/A1
A/swine/Hanoi/825/2014(H1N2)_May/A2
A/swine/Hanoi/826/2014(H1N2)_May/A4
77
A/swine/Hanoi/830/2014(H1N2)_May/A6
A/swine/Hanoi/833/2014(H1N2)_May/A6
A/swine/Hanoi/836/2014(H1N2)_May/A5
A/swine/Hanoi/888/2014(H1N2)_May/B4
A/swine/Hanoi/896/2014(H1N2)_May/C11
A/swine/Hanoi/900/2014(H1N2)_May/C11
TR
0.02
