# Supplementary material for: Swine influenza viruses in Northern Vietnam in 2013–2014
Source: Emerg Microbes Infect. 2018 Jul 2;7:123. doi: 10.1038/s41426-018-0109-y (PMC6028489; doi:10.1038/s41426-018-0109-y)
Supplement: Supplementary file 7 — Supplementary Figure S7 [file 41426_2018_109_MOESM7_ESM.pdf]

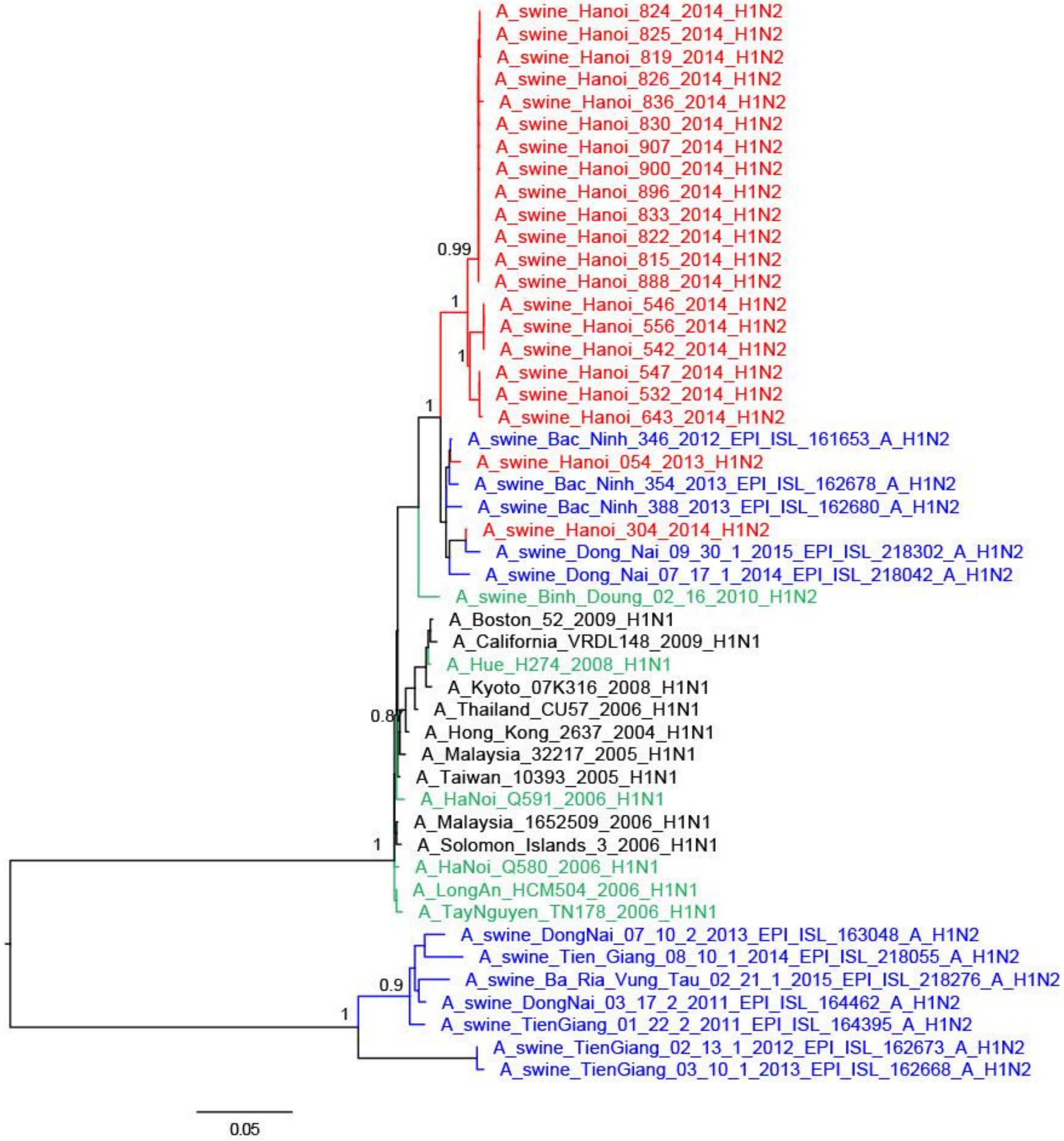

Supplementary Figure S7. Phylogenetic tree for the full length HA of H1N2 viruses isolated in a slaughterhouse in Vietnam in 2013-2014

Trees were constructed with PhyML. Branch support aLRT statistics were shown at major nodes with values larger or equal to 0.8. GenBank accession numbers of retrieved sequences are indicated. Red: sequences from our study; Blue: sequences from Takemae et al. (Ref. 28); Green: viruses from Vietnam from other studies.
