# Supplementary material for: Swine influenza viruses in Northern Vietnam in 2013–2014
Source: Emerg Microbes Infect. 2018 Jul 2;7:123. doi: 10.1038/s41426-018-0109-y (PMC6028489; doi:10.1038/s41426-018-0109-y)
Supplement: Supplementary file 8 — Supplementary Figure S8 [file 41426_2018_109_MOESM8_ESM.pdf]

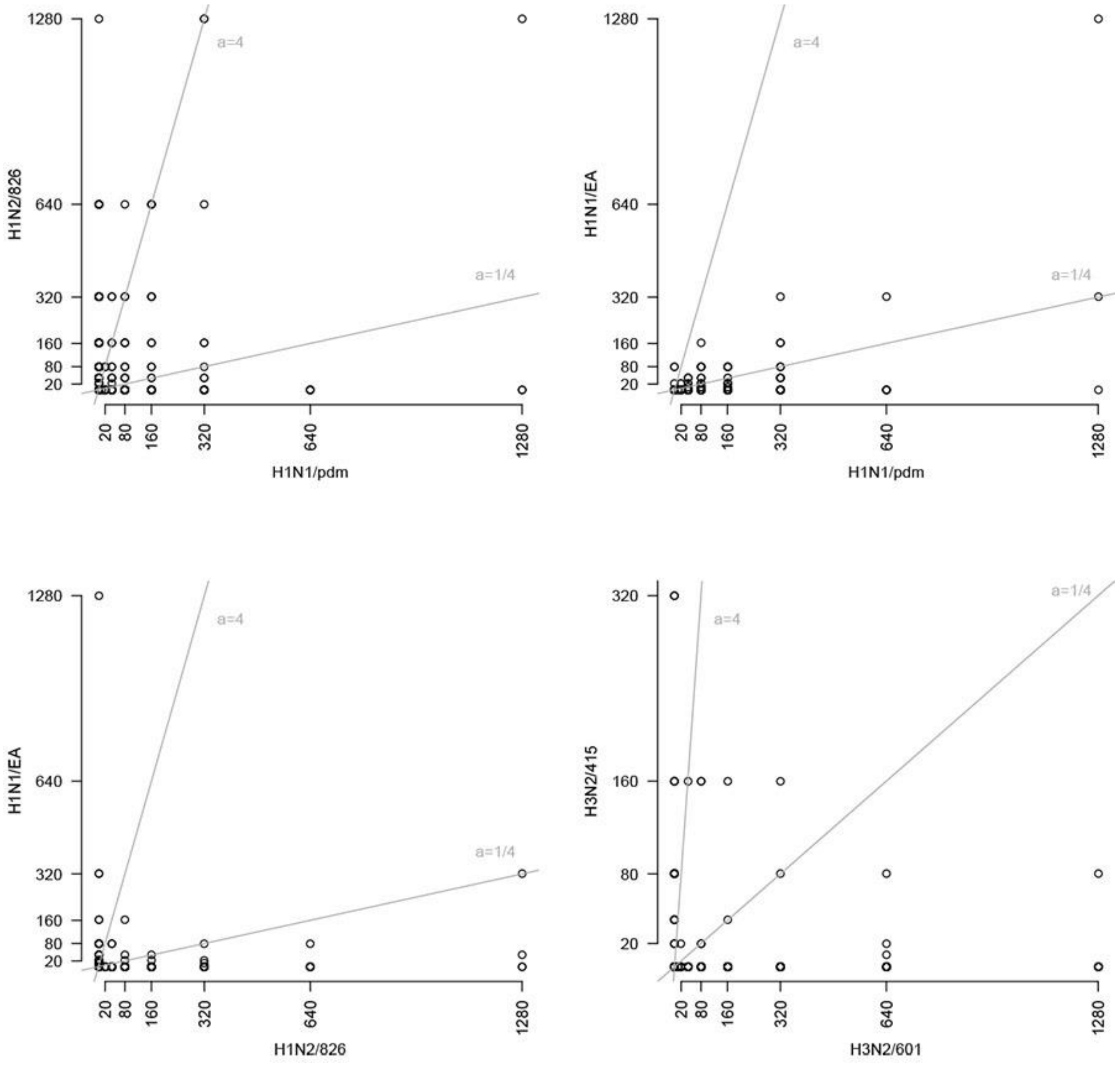

Supplementary Figure S8. Potential serological cross-reactions by haemagglutination inhibition test for viruses of same subtype

All HI positive sera are included. Sera with titers located below the line of slope a=1/4 are considered positive for the strain which titers are represented in the X axis and cross-reactive for the strain which titers are represented in the Y axis, and inversely for sera located above the line of slope a=4. Sera located in between the two lines reacted to both strains and a main strain cannot be determined (H1 “undetermined”).
